# Cytokine storm–based mechanisms for extrapulmonary manifestations of SARS-CoV-2 infection

**DOI:** 10.1172/jci.insight.166012

**Published:** 2023-05-22

**Authors:** Maria Del Nogal Avila, Ranjan Das, Joubert Kharlyngdoh, Eduardo Molina-Jijon, Hector Donoro Blazquez, Stéphanie Gambut, Michael Crowley, David K. Crossman, Rasheed A. Gbadegesin, Sunveer S. Chugh, Sunjeet S. Chugh, Carmen Avila-Casado, Camille Macé, Lionel C. Clement, Sumant S. Chugh

**Affiliations:** 1 Glomerular Disease Therapeutics Laboratory, Department of Internal Medicine, Rush University Medical Center, Chicago, Illinois, USA.; 2 Genomics Core Lab, University of Alabama at Birmingham, Birmingham, Alabama, USA.; 3 Division of Nephrology, Department of Pediatrics, Duke University Medical Center, Durham, North Carolina, USA.; 4 Department of Anatomical Pathology, Toronto General Hospital, University of Toronto, Toronto, Ontario, Canada.; 5 Instituto Nacional de Cardiología, Mexico City, Mexico.

**Keywords:** Nephrology, Chronic kidney disease, Cytokines

## Abstract

Viral illnesses like SARS-CoV-2 have pathologic effects on nonrespiratory organs in the absence of direct viral infection. We injected mice with cocktails of rodent equivalents of human cytokine storms resulting from SARS-CoV-2/COVID-19 or rhinovirus common cold infection. At low doses, COVID-19 cocktails induced glomerular injury and albuminuria in zinc fingers and homeoboxes 2 (*Zhx2*) hypomorph and *Zhx2^+/+^* mice to mimic COVID-19–related proteinuria. Common Cold cocktail induced albuminuria selectively in *Zhx2* hypomorph mice to model relapse of minimal change disease, which improved after depletion of TNF-α, soluble IL-4Rα, or IL-6. The *Zhx2* hypomorph state increased cell membrane to nuclear migration of podocyte ZHX proteins in vivo (both cocktails) and lowered phosphorylated STAT6 activation (COVID-19 cocktail) in vitro. At higher doses, COVID-19 cocktails induced acute heart injury, myocarditis, pericarditis, acute liver injury, acute kidney injury, and high mortality in *Zhx2^+/+^* mice, whereas *Zhx2* hypomorph mice were relatively protected, due in part to early, asynchronous activation of STAT5 and STAT6 pathways in these organs. Dual depletion of cytokine combinations of TNF-α with IL-2, IL-13, or IL-4 in *Zhx2^+/+^* mice reduced multiorgan injury and eliminated mortality. Using genome sequencing and CRISPR/Cas9, an insertion upstream of *ZHX2* was identified as a cause of the human *ZHX2* hypomorph state.

## Graphical abstract

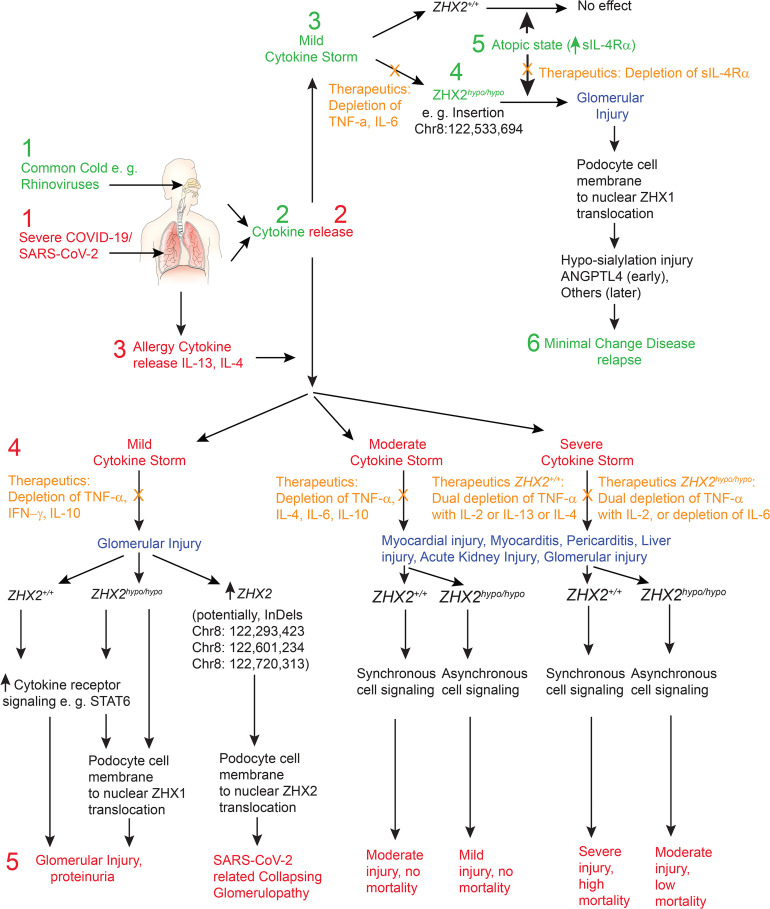

## Introduction

A striking feature of the COVID-19 pandemic is multisystem involvement (1, 2) including the respiratory tract, kidney, brain, liver, heart, gastrointestinal tract, eyes, and many other organs. The virus is not always detected in affected organs (3–6), and its presence or absence in cardiac autopsy studies does not appear to influence the extent of inflammatory cell infiltration (5). Animal models using direct SARS-CoV-2 infection have not clearly replicated this plethora of extrapulmonary manifestations. Viral infections trigger cytokine production as part of the innate and adaptive immune response. We suspected that the extensive cytokine storm documented early in the pandemic (1) may be involved in organ damage and developed novel evidence-based models of cytokine-mediated end organ damage from published human studies (1, 2) to study some of the extrapulmonary manifestations of SARS-CoV-2 infection. Of the 3 organs we studied, the literature on cardiac involvement shows elevated cardiac troponin I levels (mimicking an acute myocardial infarction), myocarditis, myocardial necrosis, pericarditis, arrhythmias, and heart failure (4, 7). Evidence of liver injury includes increased aminotransferase levels, hepatocyte injury, inflammation, and steatosis (8). Kidney manifestations are very common in hospitalized COVID-19 patients, with 38% to 65% of patients developing proteinuria (9–11), of varying degrees and duration, and 5% to 36% developing acute kidney injury (AKI) (9–12). Kidney biopsy studies in COVID-19 patients with severe, persistent proteinuria and/or kidney dysfunction have most commonly documented the collapsing variant of focal and segmental glomerulosclerosis (FSGS) and AKI (13, 14). Despite searches for viral particles in early autopsy studies (15), kidney biopsies from living patients did not reveal any viral particles (13, 14).

The advantage of building a COVID-19 cytokine storm model on kidney disease is a potential mechanistic comparison with rare manifestations of a common cold cytokine storm (16–25), with which it shares some components. Common colds, frequently caused by rhinoviruses (16), trigger 52% of relapses preceded by a defined event in patients with minimal change disease (MCD) or steroid-dependent nephrotic syndrome. While this relapse pathway is unpublished, we have considered cytokine storm to play a leading role. Since the COVID-19 cytokine storm is broader than its common cold counterpart, subtractive analysis could identify key players in specific aspects of each disease. Human and experimental MCD and most forms of FSGS are associated with low podocyte expression of transcriptional factor zinc fingers and homeoboxes 2 (ZHX2) (27). By contrast, experimental evidence suggests the collapsing variant of FSGS has high underlying podocyte ZHX2 expression (27). Contrasting the predominantly nuclear expression of ZHX proteins in kidney tubular cells, hepatocytes, cardiomyocytes, and other cells, podocytes express the majority of all ZHX proteins in a cell membrane distribution. In the right combinations and the setting of altered ZHX2 expression, systemic cytokine release could induce migration of ZHX proteins from normal (aminopeptidase A, APA; ephrin-B1) (27) or putative alternative cell membrane anchors into the podocyte nucleus. Once we obtained proof of concept using *Zhx2^fl/fl^* and stomatin family member *NPHS2* promoter–driven Cre (*NPHS2 promoter–Cre^+/+^*) mice, we used *BALB/cJ* mice, an established model of the *Zhx2* hypomorph state (27–33), and *BALB/c* mice (*Zhx2^+/+^*) to illustrate how *Zhx2* expression affects cytokine storm–related morbidity and mortality. This allowed us to dissect disease mechanisms and develop therapeutic approaches to reduce cytokine storm–related end organ damage.

## Results

### Developing Common Cold and COVID-19 cytokine storm cocktails.

**Figure 1:**
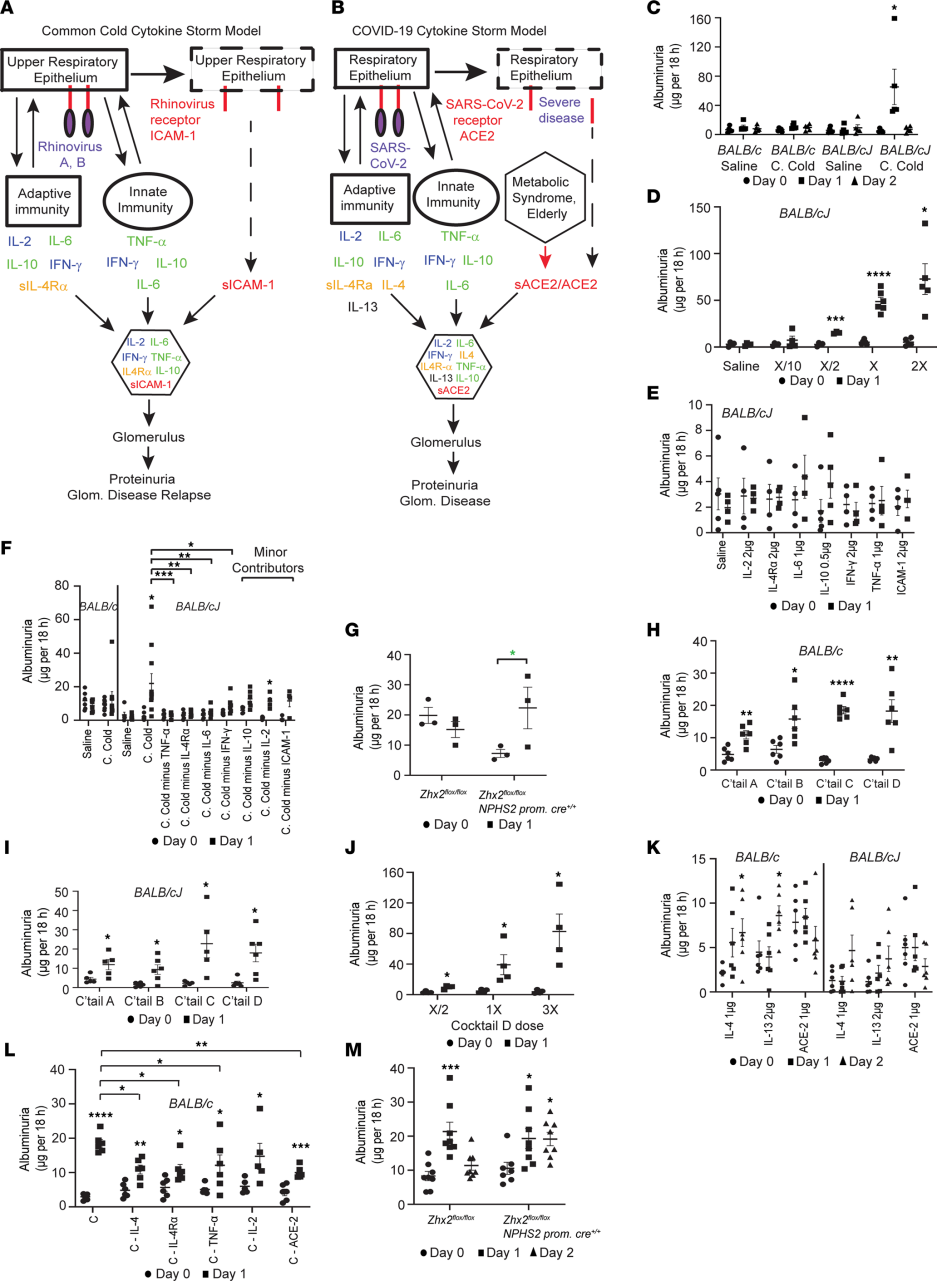
Development of cytokine storm models. (**A**) Schematic representation of rhinovirus Common Cold (CC) model. ACE2, angiotensin-converting enzyme 2; ICAM-1, intercellular adhesion molecule 1; sACE2 and sIL-4Rα, soluble variants of transmembrane proteins. (**B**) Schematic representation of COVID-19 cytokine model. (**C**) Albuminuria following CC cocktail dose X or control saline in *BALB/cJ* and *BALB/c* mice (*n* = 5 mice/group). (**D**) Dose-response effect of CC cocktail on albuminuria in *BALB/cJ* mice (*n* = 4 – 6 mice/group). (**E**) Albuminuria following injection of individual CC cocktail components dose X in *BALB/cJ* mice (*n* = 4–5 mice/group). (**F**) Albuminuria after injecting CC cocktail dose X/2, or X/2 minus individual components, in *BALB/cJ* mice (*n* = 4 to 11 mice/group). BALB/c mice do not develop albuminuria at dose X/2. (**G**) CC cocktail dose X/15 induced albuminuria in podocyte-specific *Zhx2*-deficient *Zhx2^fl/fl^ NPHS2 promoter–Cre^+/+^* and control *Zhx2^fl/fl^* mice (*n* = 3 mice/group). (**H**) COVID-19 cocktail dose X/2 induced albuminuria in *BALB/c* mice (*n* = 6 mice/group). (**I**) COVID-19 cocktail dose X/2 induced albuminuria in *BALB/cJ* mice (*n* = 5–6 mice/group). (**J**) Dose-response effect of Cocktail D on albuminuria in *BALB/cJ* mice (*n* = 4 mice/group). (**K**) Albuminuria in *BALB/c* and *BALB/cJ* mice after injecting individual components (dose X) of COVID-19 cocktails (*n* = 6 mice/group). (**L**) Albuminuria after injecting Cocktail C dose X/2 or Cocktail C dose X/2 minus individual components that target podocytes in BALB/c mice (*n* = 5–6 mice/group). (**M**) Cocktail C dose X/5 induced albuminuria in *Zhx2^fl/fl^ NPHS2 promoter–Cre^+/+^* and control *Zhx2^fl/fl^* mice (*n* = 7–8 mice/group; age 18 weeks). * *P* < 0.05; ** *P* < 0.01; *** *P* < 0.001; **** *P* < 0.0001, 1-way Anova (Dunnett, **C**, **K**, **M**; FDR, method of Benjamini, Krieger, and Yekutieli [FDR-BKY], **F**, **L**), multiple *t* test comparisons (Holm-Šídák, **D**, **E**, **F**, **H**, **I**, **J**; FDR-BKY, **L**), and simple *t* test, 1 tailed (green asterisk, **G**). Data represent mean ± SEM.

The potentially novel Common Cold cocktail was designed from published literature (16–25) to mimic MCD and FSGS relapse after a common cold (Figure 1A). Since chronic atopy, present in up to half of patients with MCD patients (24), is associated with increased expression of transmembrane and soluble IL-4Rα splice variants (25), IL-4Rα was included in the Common Cold cocktail. Because circulating levels of the rhinovirus A, B receptor ICAM-1 levels are increased during common colds (20, 21), ICAM-1 was included in the Common Cold cocktail. COVID-19 Cocktails A to D were developed in a stepwise manner to model hospitalized COVID-19 patients needing intensive care (Figure 1B and Table 1). The first 5 cytokines (Table 1) are common to all cocktails. Circulating IL-4Rα levels were increased in COVID-19 patients with proteinuria when compared with general COVID-19–positive patients or their age-, race-, and sex-matched nondisease controls (Supplemental Figure 1A; supplemental material available online with this article; https://doi.org/10.1172/jci.insight.166012DS1). ACE2, the COVID-19 receptor, was included in COVID-19 cocktails since plasma sACE2 levels are significantly higher in COVID-19 patients in intensive care (34, 35) and in elderly and metabolic syndrome patients who are predisposed to severe COVID-19 disease (36). High plasma IL-13 and IL-4 in patients with COVID-19 requiring intensive care (1) indicates acute activation of the allergy cytokine pathway in this disease. Removing ICAM-1 in the Common Cold cocktail and adding sACE2 resulted in COVID-19 Cocktail A. Removing sIL-4Rα from Cocktail A and adding IL-4 and IL-13 made Cocktail B, whereas adding IL-4 to Cocktail A gave Cocktail C. Adding IL-13 to Cocktail C gave Cocktail D.

Common Cold cocktail dose X induced acute albuminuria in *Zhx2* hypomorph *BALB/cJ* but not in Zhx2^+/+^ *BALB/c* mice (Figure 1C). A dose-response study showed X/2 to be the threshold nephritogenic dose in *BALB/cJ* mice (Figure 1D) that also induced histological changes on electron microscopy (Supplemental Figure 1B). Individually, none of the cytokines injected in the same dose as combination X induced albuminuria (Figure 1E). Eliminating individual cytokines helped identify major (albuminuria significantly lower than with complete cocktail) and minor synergistic contributors (albuminuria lower but not statistically different from complete cocktail) in *BALB/cJ* mice (Figure 1F). Lower baseline albuminuria in *BALB/cJ* versus *BALB/c* mice (Figure 1F) is previously published (27). As a further proof of concept, the Common Cold cocktail induced albuminuria in podocyte-specific *Zhx2^fl/fl^ NPHS2* *promoter*–*Cre^+/+^* mice but not in *Zhx2^fl/fl^* mice (Figure 1G). Lower baseline albuminuria in young *Zhx2^fl/fl^ NPHS2 promoter*–*Cre^+/+^* compared with *Zhx2^fl/fl^* mice is also previously described (27). Buffalo Mna rats with active FSGS also developed worsening of proteinuria after injection of a rat Common Cold cytokine cocktail (Supplemental Figure 1C), mimicking disease worsening after a common cold.

All COVID-19 cocktails induced albuminuria in *BALB/c* (Figure 1H) and *BALB/cJ* (Figure 1I) mice. A dose-response study with these cocktails (e.g., Cocktail D in *BALB/cJ* mice, Figure 1J) showed X/2 to be the threshold nephritogenic dose. Replacement of sICAM-1 (Common Cold cocktail) with sACE2 (Cocktail A) induced significant albuminuria in *BALB/c* mice (Figure 1H). Cocktails C and D tended to induce more albuminuria in *BALB/cJ* mice. Cytokines unique to COVID-19 cocktails (IL-4, IL-13), but not sACE2, induced mild albuminuria when injected alone in *BALB/c* mice (Figure 1K). Unlike the Common Cold cocktail, removing single components that interact with podocytes from Cocktail C reduced but did not eliminate albuminuria (Figure 1L), suggesting more complex synergy in the pathogenesis of glomerular injury by COVID-19 cocktails. COVID-19 cocktails induced prominent histological changes in mouse glomeruli on electron microscopy (Supplemental Figure 1D), whereas light microscopy was unremarkable. No cocktail increased serum creatinine (Supplemental Figure 1E) at dose X/2 in *BALB/c* or *BALB/cJ* mice. Finally, Cocktail C induced acute albuminuria in both podocyte-specific *Zhx2^fl/fl^ NPHS2* *promoter*–C*re^+/+^* and *Zhx2^fl/fl^* mice, but albuminuria persisted longer in the podocyte *Zhx2* hypomorph state (Figure 1M).

### Systemic manifestations of synergistic multicytokine injury induced by COVID-19 cocktails.

**Figure 2:**
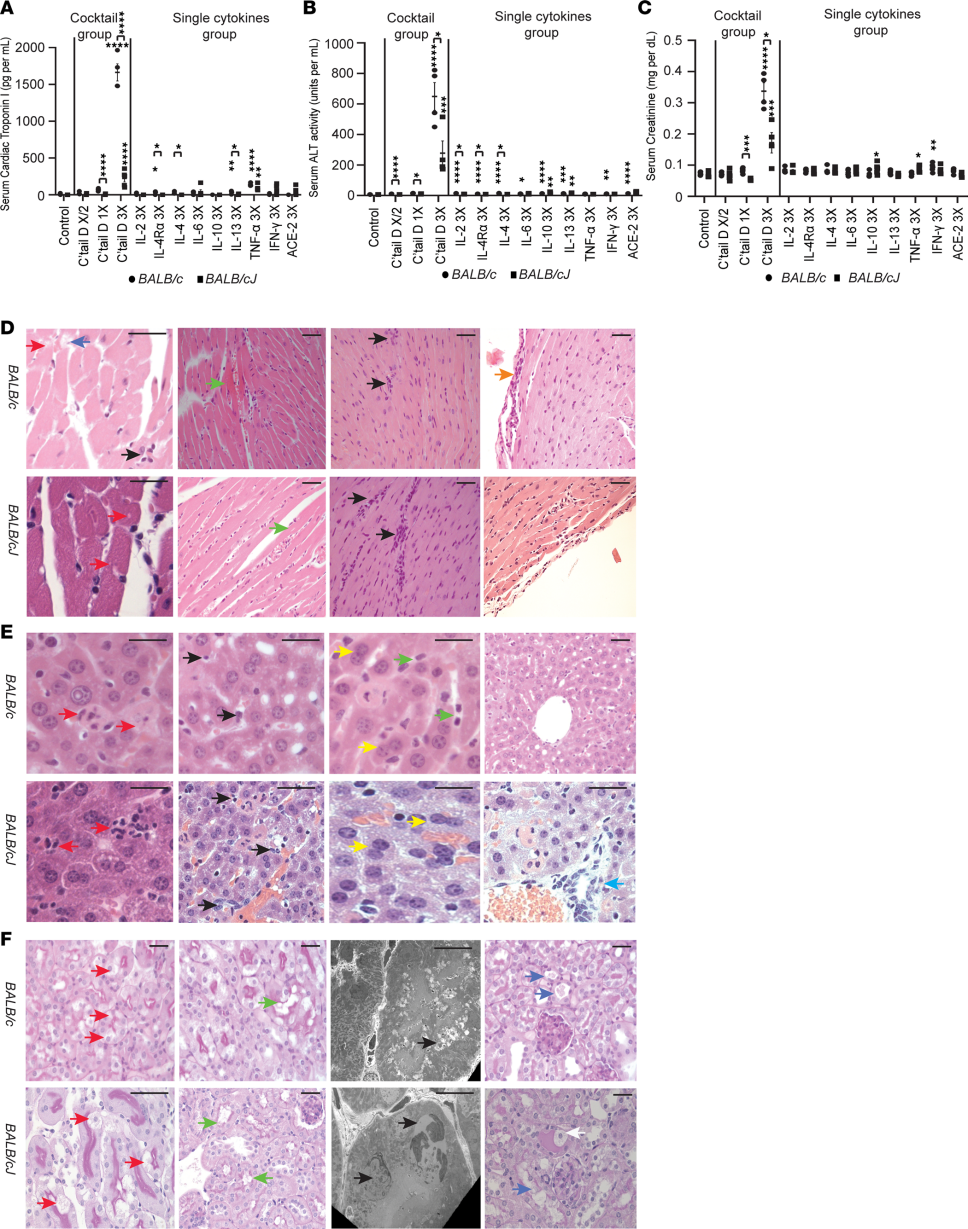
Comparison of systemic injury induced by high dose (3×) of Cocktail D and lower doses or individual components in high doses in *BALB/c* and *BALB/cJ* mice. Data represent mean ± SEM. (**A**) Acute myocardial injury assessed by serum cTPI3 levels (*n* = 4–6 mice/group). (**B**) Acute liver injury assessed by serum ALT activity levels (*n* = 4–6 mice/group). (**C**) AKI assessed by serum creatinine levels measured using mass spectrometry (*n* = 4–6 mice/group). (**D**) Histological characterization of acute cardiac injury (*n* = 3 mice/group) using H&E-stained sections in Cocktail D dose 3×–injected mice. Myocytolysis (red arrows), inflammation (black arrows), fibril disruption (blue arrows), hypereosinophilia (green arrows), and pericarditis (orange arrow). (**E**) Histological characterization of acute liver injury (*n* = 3 mice/group) using H&E-stained sections in Cocktail D dose 3×–injected mice. Hepatocellular injury (red arrows), inflammation (black arrows), prominent Kupffer cells (green arrows), regenerative changes (yellow arrows), and peri-central vein injury (blue arrow). (**F**) Histological assessment of AKI (*n* = 3 mice/group) using periodic acid–Schiff–stained sections (columns 1, 2, 4) and electron microscopy (column 3) (Leica Microsystems) in Cocktail D dose 3×–injected mice. First 3 columns show proximal tubules, last column shows distal tubules. In proximal tubules, vacuolation (red arrows), brush border disruption (green arrows) and tubular degeneration (black arrows) were noted. In distal tubules, evidence of desquamation (blue arrows) was present. Foam cells were also noted (white arrows). Electron microscopy scale bars: *BALB/c*, 2.66 μm; *BALB/cJ*, 2 μm. Light microscopy scale bars: 20 μm. * *P* < 0.05; ** *P* < 0.01; *** *P* < 0.001; **** *P* < 0.0001, determined by 1-way ANOVA (Dunnett, panels **A**–**C**) and multiple *t* test comparisons (Holm-Šídák, panels **A**–**C**), with cocktail and single-cytokine groups analyzed in parallel.

Injection of higher nephritogenic doses (3×) of Cocktail D induced more albuminuria (Figure 1J) but also caused several-fold elevation of serum cardiac troponin I type 3 (cTPI3; myocardial injury, Figure 2A), serum alanine aminotransferase (ALT; acute liver injury, Figure 2B), serum creatinine (AKI, Figure 2C), and plasma creatine kinase (CK; skeletal muscle injury, Supplemental Figure 2A). cTPI3, ALT, and albuminuria also increased at 3× dose for some individual cytokines, albeit at a significantly lower level than the cocktail (Figure 2, A and B, and Supplemental Figure 2, B–E). Timed urine collection in metabolic cages for albuminuria assessment was not conducted for Cocktail D 3× dose–injected *BALB/c* mice in view of high mortality (see below). Since most cells other than podocytes (e.g., kidney tubular cells, hepatocytes, cardiomyocytes) express ZHX proteins in a predominantly nuclear pattern, we tested whether low constitutive nuclear ZHX2 expression affected the outcome of multiorgan cytokine injury by comparing *BALB/c* with *BALB/cJ* mice. Cocktail D 3× dose induced substantially more severe cardiac, liver, and acute kidney injury in *BALB/c* compared with *BALB/cJ* mice, suggesting that the latter are protected by the *Zhx2* hypomorph state. While liver, kidney, and glomerular *Zhx2* hypomorph state in *BALB/cJ* mice is previously described (27–29), we found similar changes in the heart and skeletal muscle (Supplemental Figure 2F). mRNA expression of cytokine receptors, ACE2, other ZHX proteins, and select signaling pathway proteins in heart, liver, skeletal muscle, and glomeruli was similar between *BALB/cJ* and *BALB/c* *mice* (Supplemental Figure 2, G–J; exceptions, higher *Ace2* in *BALB/cJ* glomeruli, higher *Stat5* in *BALB/c* skeletal muscle). Cardiac histology (Figure 2D) revealed myocytolysis, focal fibrillar disruption and hypereosinophilia, inflammation (myocarditis), and pericarditis. Liver histology (Figure 2E) showed substantial hepatocellular injury, prominent Kupffer cells, frequent degenerative and regenerative changes, and mild inflammation. Histological evaluation of the kidney tubulointerstitial compartment (Figure 2F) revealed evidence of proximal tubular injury as frequent vacuolation, luminal widening, brush border disruption, and tubular epithelial cell desquamation. Epithelial cell desquamation, presence of foam cells, and vacuolation were also noted in distal tubules. Glomerular injury was prominent in both strains, with extensive foot process effacement in *BALB/c* (Supplemental Figure 2K) and multifocal effacement of podocyte foot processes in *BALB/cJ* (Supplemental Figure 2L) mice. Several areas of glomerular basement membrane remodeling were noted in both strains. Skeletal muscle histology showed focal inflammation (Supplemental Figure 2M). Morphometric differences in these organs 24 hours after injection of Cocktail D 3× were noted between *BALB/c* and *BALB/cJ* mice (Supplemental Figure 2, N–P). However, there was no evidence of severe or extensive inflammation.

### Therapeutic cytokine depletion in mild cytokine storms.

**Figure 3:**
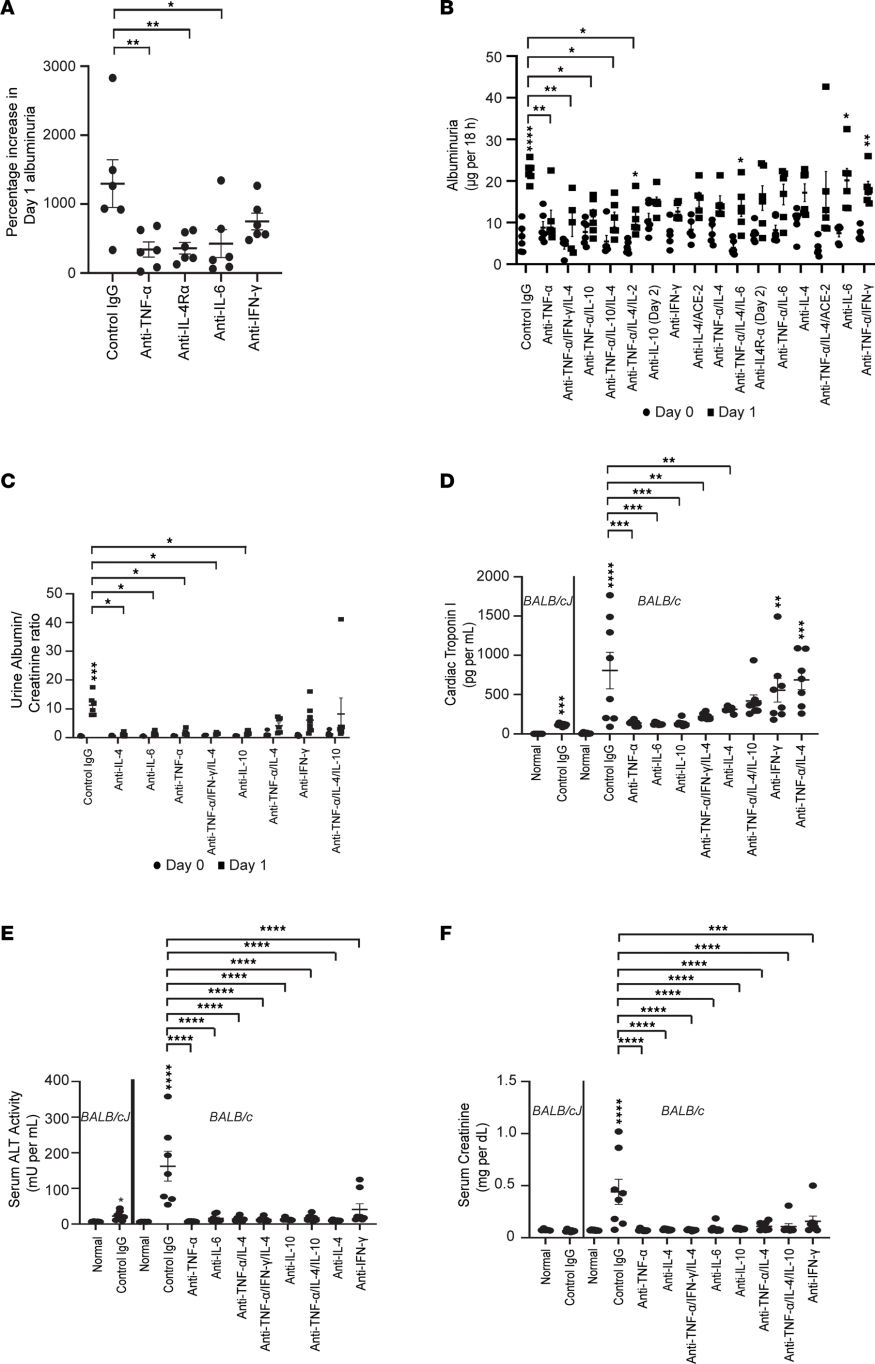
Therapeutic strategies for mild and moderate cytokine storms in glomerular and systemic disease. All depleting antibodies or control IgG were injected intravenously 1 hour after model induction. Data represent mean ± SEM. (**A**) Composite graph of multiple studies showing percentage increase in 18-hour albuminuria from baseline on day 1 in the Common Cold cocktail X/2 model, with injection of control IgG or depleting antibodies against single cocktail components (*n* = 6 *BALB/cJ* mice/group). (**B**) Albuminuria at baseline and on day 1 after injection of Cocktail C X/2 followed by control IgG or depleting antibodies (*n* = 5–6 *BALB/c* mice/group). (**C**) Urine albumin/creatinine ratio at baseline and day 1 after injecting Cocktail D 1.8× followed by control IgG or 1 or more depleting antibodies (*n* = 5–8 *BALB/c* mice/group). (**D**) Serum cTPI3 levels on day 1 after injecting Cocktail D 1.8× followed by control IgG or 1 or more depleting antibodies (*n* = 5–8 *BALB/c* mice/group). Control and Cocktail D 1.8× + IgG–injected *BALB/cJ* mice shown for comparison. (**E**) Serum ALT activity on day 1 after injecting Cocktail D 1.8× followed by control IgG or 1 or more depleting antibodies (*n* = 5–8 *BALB/c* mice/group). Control and Cocktail D 1.8× + IgG–injected BALB/cJ mice shown for comparison. (**F**) Serum creatinine on day 1 after injecting Cocktail D 1.8× followed by control IgG or 1 or more depleting antibodies (*n* = 5–8 *BALB/c* mice/group). Control and Cocktail D 1.8× + IgG–injected *BALB/cJ* mice are shown for comparison. * *P* < 0.05; ** *P* < 0.01; *** *P* < 0.001; **** *P* < 0.0001, determined by 1-way ANOVA (Dunnett, **A**–**F**) and multiple *t* test comparisons (Holm-Šídák, **B** and **C**).

Since common colds cause mild cytokine storms, glomerular injury therapeutic studies were conducted using low-dose cocktails (1× or less). Also, low cocktail dose induced albuminuria without creatinine elevation due to superimposed AKI. Antibody-mediated depletion of TNF-α, IL-6, and IL-4Rα after common cold model induction in *BALB/cJ* mice resulted in a significant decline in albuminuria (Figure 3A), suggesting potential therapeutic avenues for treating relapse of primary glomerular diseases after a common cold. Injecting Cocktail C X/2 (Figure 3B), followed by single or combination cytokine depletion, in *BALB/c* mice showed significant reduction in albuminuria by anti–TNF-α antibody and select anti–TNF-α antibody–based combinations that included anti–IL-4, anti–IL-10, and anti–IFN-γ antibodies. In many cases, depleting more cytokines was not always better, suggesting that overmanipulation of the cytokine milieu can be counterproductive.

### Therapeutic cytokine depletion in moderate cytokine storms.

An intermediate-dose Cocktail D 1.8× model was developed in *BALB/c* mice to mimic hospitalized COVID-19 patients with systemic manifestations beyond proteinuria but not requiring intensive care and also helped us understand the evolution of injury beyond mild cytokine storms. The use of anti–IL-4, anti–IL-6, anti–TNF-α, anti–IL-10, and an anti–TNF-α antibody–based combination was effective in reducing albuminuria (Figure 3C) and cTPI3 (Figure 3D), whereas all regimens improved serum ALT levels (Figure 3E) and normalized serum creatinine (Figure 3F). Additional studies using anti–TNF-α antibody–based combinations in *BALB/cJ* mice using the Cocktail D X/2 model (Supplemental Figure 2Q) and cytokine receptor blockage using the Cocktail C X/2 model in *BALB/c* mice (Supplemental Figure 2R) also showed significant improvement in albuminuria. Morphometric analysis of these studies showed significant improvement in histological changes (pericarditis with microcalcifications, myocarditis, myocardial injury, acute hepatic injury, AKI, podocyte foot process effacement) in the most effective regimens discussed (Supplemental Figure 3).

### Therapeutic cytokine depletion to prevent mortality and reduce multiorgan toxicity in severe cytokine storms.

**Figure 4:**
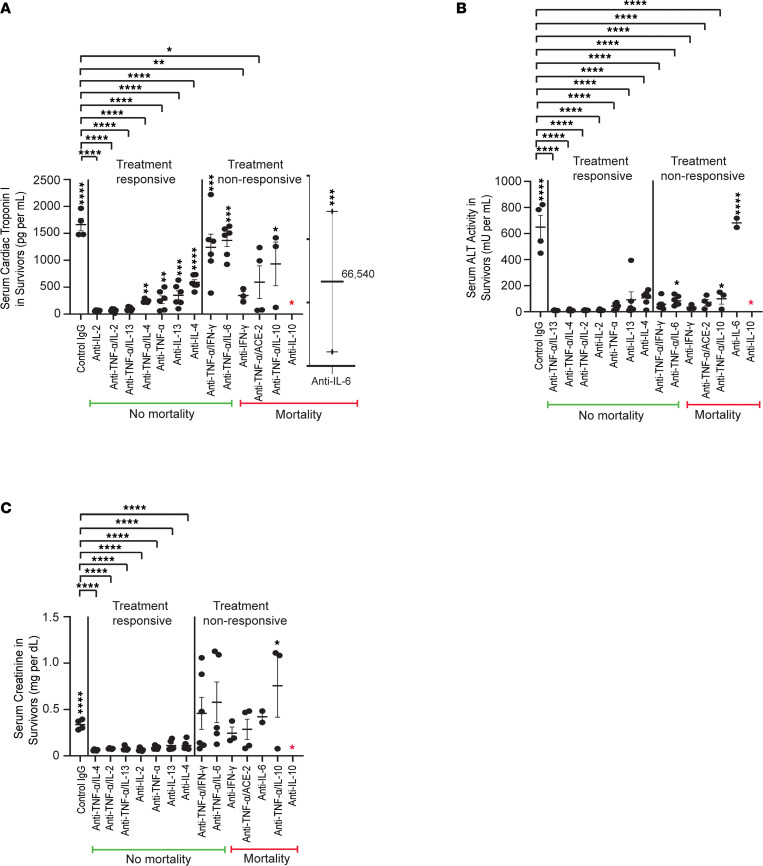
Therapeutic strategies for severe cytokine storms in systemic disease in *BALB/c* mice. Number of mice injected per group is shown in Table 2. All depleting antibodies or control IgG were injected intravenously 1 hour after model induction. Data represent mean ± SEM. Red asterisk indicates universal mortality. Since mortality was higher with metabolic cage use (5/6) than without (2/6) in the control IgG group, timed urine collection for albuminuria was not conducted in these studies. (**A**) Serum cTPI3 levels on day 1 among survivors after injecting Cocktail D 3× followed by control IgG or 1 or more depleting antibodies. (**B**) Serum ALT activity levels on day 1 among survivors after injecting Cocktail D 3× followed by control IgG or 1 or more depleting antibodies. (**C**) Serum creatinine levels on day 1 among survivors after injecting Cocktail D 3× followed by control IgG or 1 or more depleting antibodies. * *P* < 0.05; ** *P* < 0.01; *** *P* < 0.001; **** *P* < 0.0001, determined by 1-way Anova (Dunnett, panels **A**–**C**).

Injecting Cocktail D 3× in *BALB/c* mice caused high mortality at 24 hours (Table 2) and modeled patients with COVID-19 requiring intensive care. Metabolic cages for urine collection were not used in this study to avoid near-universal mortality (5/6 dead) in the control IgG group. Depletion of TNF-α in combination with IL-2, IL-13, or IL-4, and single depletion of TNF-α, IL-13, IL-2, or IL-4, were most effective in disrupting cocktail component synergy; eliminated mortality; and normalized overall activity in mice at 24 hours (treatment-responsive groups, Table 2). These interventions, especially specific anti–TNF-α antibody–based combinations, were most efficacious in reducing serum levels of cTPI3, ALT, and creatinine (Figure 4, A–C). Monotherapy for IL-2 depletion reduced organ injury effectively, but mice still had some standing hair at 24 hours, indicating distress. Monotherapy for depletion of IL-6, IL-10, and IFN-γ and several combinations were counterproductive (treatment-nonresponsive groups, Table 2). Morphometric analysis of heart, liver, and kidney showed significant improvement with the most efficacious regimens (Supplemental Figure 4).

**Figure 5:**
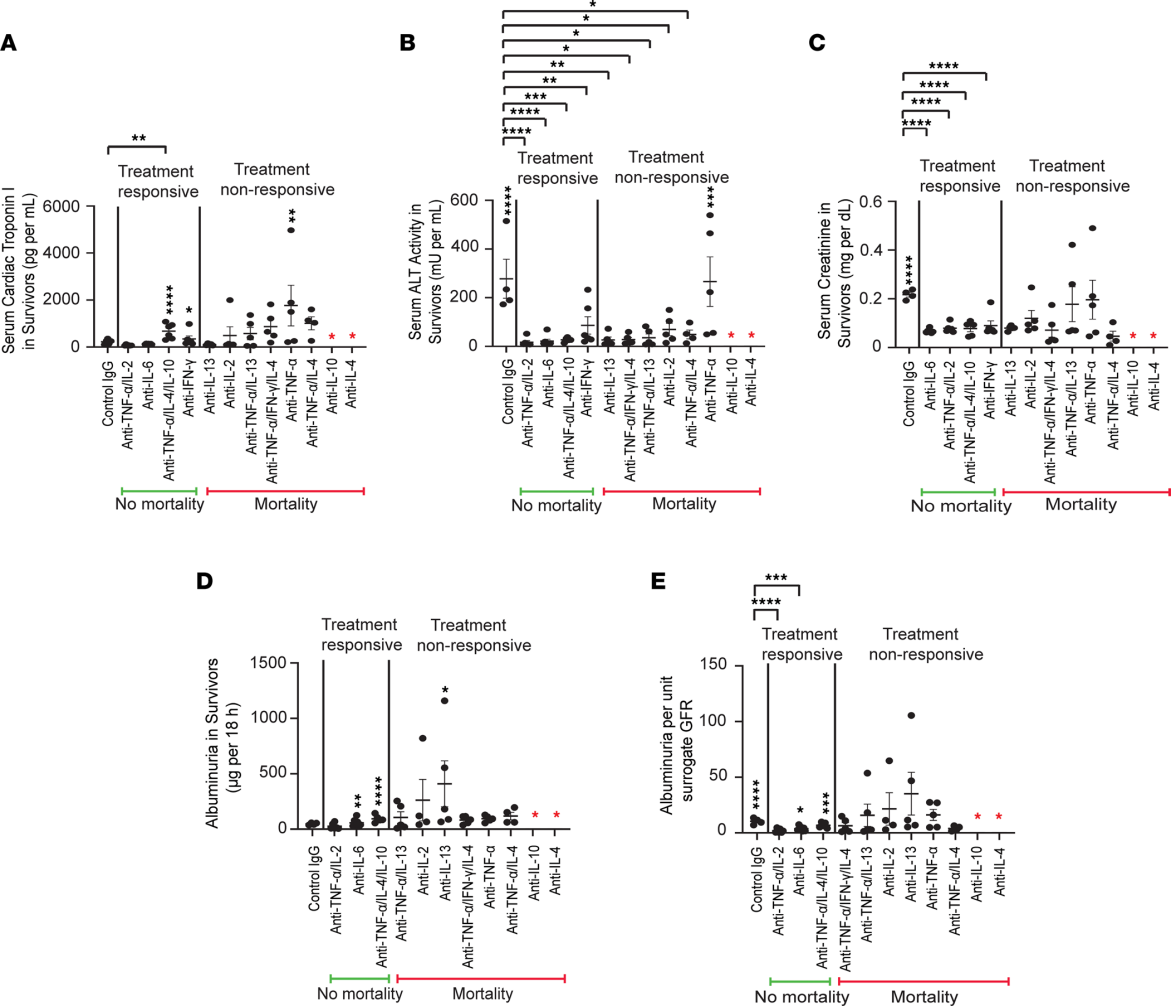
Therapeutic strategies for severe cytokine storms in systemic disease in *BALB/cJ* mice. Number of mice injected per group is shown in Table 3. All depleting antibodies or control IgG were injected intravenously 1 hour after model induction. Data represent mean ± SEM. Red asterisk indicates universal mortality. (**A**) Serum cTPI3 levels on day 1 among survivors after injecting Cocktail D 3× followed by control IgG or 1 or more depleting antibodies. (**B**) Serum ALT activity levels on day 1 among survivors after injecting Cocktail D 3× followed by control IgG or 1 or more depleting antibodies. (**C**) Serum creatinine levels on day 1 among survivors after injecting Cocktail D 3× followed by control IgG or 1 or more depleting antibodies. (**D**) The 18-hour albuminuria, unadjusted for glomerular filtration rate (GFR), among survivors after injecting Cocktail D 3× followed by control IgG or 1 or more depleting antibodies. (**E**) The 18-hour albuminuria per unit surrogate GFR (1 divided by serum creatinine) among survivors after injecting Cocktail D 3× followed by control IgG or 1 or more depleting antibodies. * *P* < 0.05; ** *P* < 0.01; *** *P* < 0.001; **** *P* < 0.0001, determined by 1-way ANOVA (Dunnett, panels **A**–**E**).

The *BALB/cJ* mouse – Cocktail D 3× model had low mortality even while using metabolic cages (Table 3), and, with one exception, therapeutic response patterns were different from *BALB/c* mice. The most effective regimen was a combination of TNF-α and IL-2 depletion (treatment-responsive regimens, Table 3; and Figure 5, A–E). Depletion of IL-6 or IFN-γ alone, or a combination of TNF-α, IL-4, and IL-10, also improved organ toxicity. By contrast, individual depletion of TNF-α was ineffective, whereas depletion of IL-4 or IL-10 induced universal mortality (treatment-nonresponsive regimens, Table 3). Morphometric comparison between these groups showed significant histological improvement in the abovementioned effective treatment groups (Supplemental Figure 5).

### Genomics of the ZHX2 hypomorph state.

**Figure 6:**
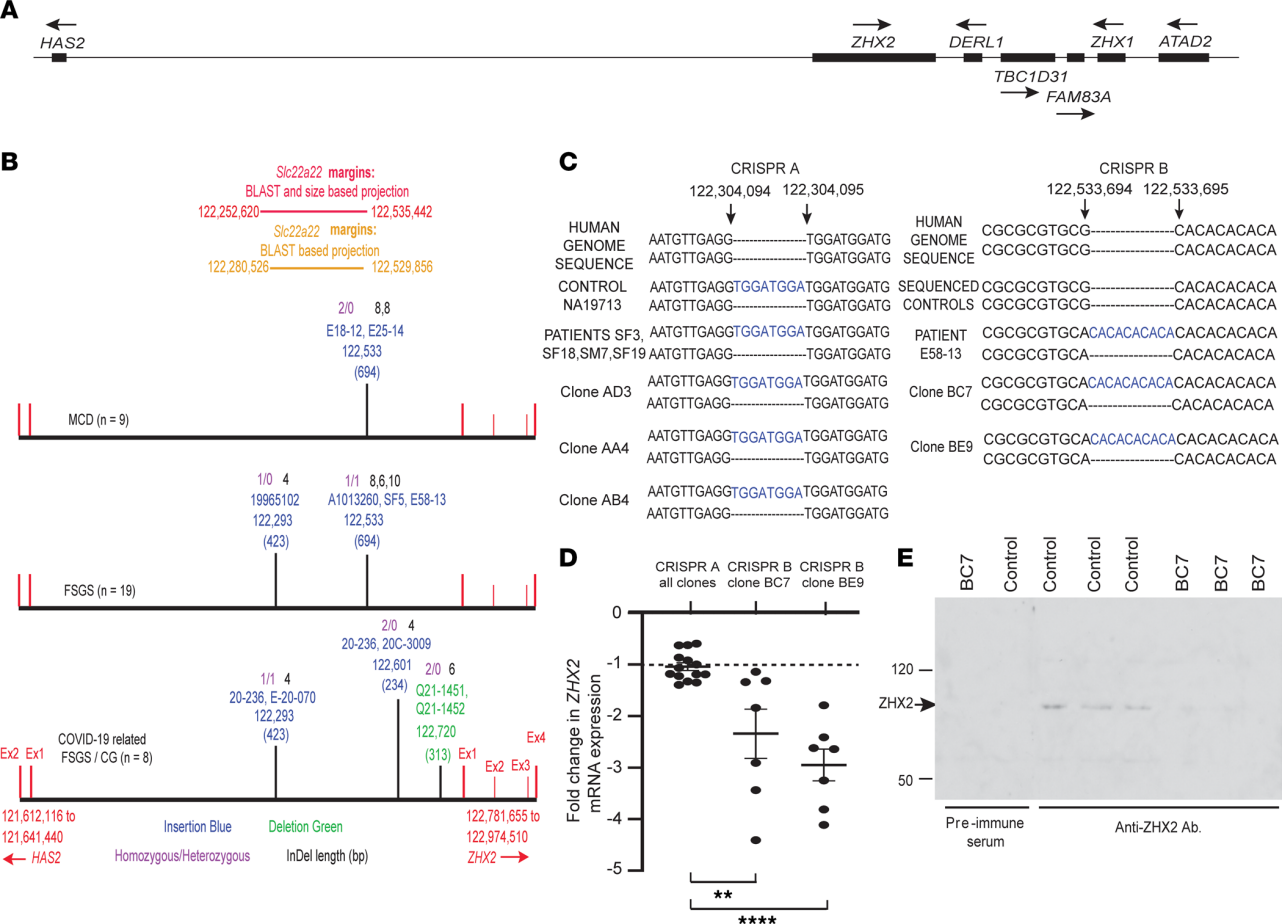
Insertions and deletions in noncoding DNA affect *ZHX2* expression in patients with glomerular disease. (**A**) Schematic representation of *ZHX2* and neighboring genes on chromosome 8. CG, collapsing glomerulopathy. (**B**) Mapping of shared insertions and deletions (InDels) among patients with MCD (*n* = 9 patients), FSGS (*n* = 19 patients), and COVID-19–related FSGS collapsing variant (*n* = 8 patients). The most common shared insertion at 122,533,694 bp could be mapped at or near the theoretical beginning of the rodent-expressed gene *Slc22a22*, defunct in humans. Another shared insertion at 122,293,423 was located near the theoretical end of *Slc22a22*. None of these InDels were noted in controls (*n* = 33) or the 1000 Genomes Project (*n* = 2,504 participants). Ex, exon. (**C**) Schematic representation of CRISPR/Cas9-assisted genome-edited clones of a single cell–derived cultured human podocyte cell line that contain an 8 bp insertion common between patients and a control participant (CRISPR A), or a 10 bp shared insertion at 122,533,694 that was absent in controls and the 1000 Genomes Project (CRISPR B). (**D**) Fold-change *ZHX2* mRNA expression in genome-modified clones (CRISPR A, 3 clones, data pooled *n* = 14 templates; study CRISPR B, 2 clones, *n* = 7 templates/clone) compared with the parent single cell–derived cultured human podocyte cell line (dotted line). Data represent mean ± SEM. (**E**) Western blot comparing ZHX2 expression in the control single cell–derived parent cell line and 1 of 2 mutant clones with insertion at 122,533,694. Numbers on left represent kilodaltons. ** *P* < 0.01; **** *P* < 0.0001, determined by 1-way ANOVA (Dunnett).

Since large-scale whole-exome sequencing studies did not identify any *ZXH2*-related disease-causing variants (37, 38), the genomes of 36 patients with glomerular disease (9 MCD, 19 FSGS, 8 COVID-19–related FSGS collapsing variant) and 33 controls (Supplemental Table 1) were sequenced from the beginning of hyaluronan synthase 2 (*HAS2*; the immediate upstream gene) (Figure 6A) to the end of *ZHX2*. The 1000 Genomes Project database (phase 3, 2,504 participants) was used as an additional control. Multiple insertions and deletions (InDels), 3 bp or larger, noted exclusively in the patient population using CLC Genomics software were validated using IGV software as a second screening method, and only InDels present by both methods were included (Figure 6B; Table 4; Supplemental Figure 6, A–C; and Supplemental Figure 7A). Six of 9 MCD patients, 10 of 19 FSGS patients, and all 8 COVID-19 CG patients had InDels. Three insertions and 1 deletion were shared by 2 or more patients (Figure 6B and Table 4). The insertion at 122,533,694 was present exclusively in patients with primary MCD, primary FSGS, or Hodgkin lymphoma FSGS tip lesion. Two patients with COVID-19 shared an insertion with a patient with FSGS, whereas 1 other insertion and 1 deletion were shared among patients with COVID-19 only. Interspecies analysis of the genome showed the presence of the gene *Slc22a22* between HAS2 and ZHX2 in mice and rats, but this gene is nonfunctional in higher species, including humans (Supplemental Figure 7B). Fine mapping and analysis of remnants of the mouse *Slc22a22* (a prostaglandin transporter) in the human genome showed the shared insertion at 122,533,694 as being present at or near the origin of this gene site (Figure 6B). None of the shared insertions were noted in patients with diabetic nephropathy, the most common glomerular disease in the Western world (Supplemental Figure 6, A and C; Supplemental Figure 8, A and B; and Supplemental Table 2). In this condition, shared InDels were upstream of *HAS2* and not *ZHX2*, though 1 was present in *ZHX2* intron 2 (Supplemental Figure 6C). No InDels were common between diabetic and nondiabetic patients, although an occasional site was shared (Supplemental Figure 8C). In addition, apolipoprotein L1 (*APOL1*) gene variants, noted in some African American patients with FSGS collapsing variant (39), were checked in a limited subset of our patients because of limited viable residual genomic DNA from biopsies. One African American patient with recurrent CG (SF19) had a G_2_/G_2_ genotype, whereas a White patient (SM2) and 2 patients with COVID-19–related collapsing glomerulopathy from Mexico City (not included in above analysis) had wild-type G_0_/G_0_ alleles.

In view of prior documentation of low podocyte ZHX2 expression in human MCD and FSGS (27), the insertion at Chr8: 122,533,694 was replicated in a single cell–derived cultured human podocyte cell line using CRISPR/Cas9 technology (study CRISPR B, Figure 6C). For comparison, another insertion noted in patients and a control (control CRISPR A) was also replicated. *ZHX2* mRNA expression was unchanged in all CRISPR A cell line clones (data pooled), and significant downregulation was noted in both clones generated for CRISPR B (Figure 6D). Reduced expression of ZHX2 protein in a CRISPR B line compared with the parent cell line was noted on Western blot (Figure 6E and Supplemental Figure 8D).

### Synchronous versus asynchronous activation of cell signaling pathways in Zhx2^+/+^ and Zhx2 hypomorph mice.

**Figure 7:**
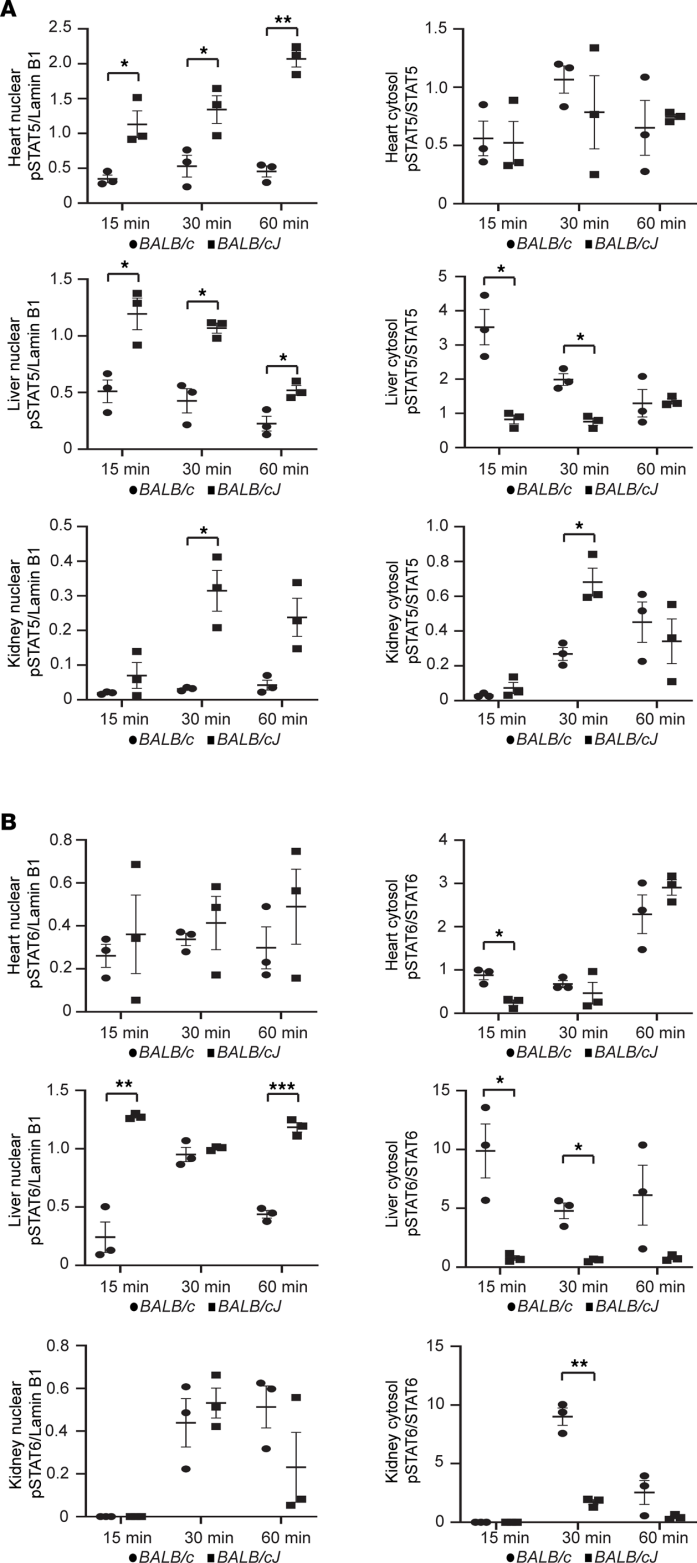
Activation of STAT5 and STAT6 signaling pathways in heart, liver, and kidney by Cocktail D. Cocktail D 3× dose–injected mice (*n* = 3 mice/group, each mouse organ assessed individually) were studied 15, 30, and 60 minutes after injection. Saline-injected mice did not activate STAT pathway signaling (data not shown). Data represent mean ± SEM. (**A**) Graphical comparison of p-STAT5 Western blot densitometry expressed as a ratio with lamin B1 (nuclear extracts) and STAT5 (cytosolic extracts) in heart, liver, and kidney between *BALB/c* (normal *Zhx2* expression) and *BALB/cJ* (low *Zhx2* expression) mice. (**B**) Graphical comparison of p-STAT6 Western blot densitometry expressed as a ratio with lamin B1 (nuclear extracts) and STAT6 (cytosolic extracts) in heart, liver, and kidney between *BALB/c* and *BALB/cJ* mice. * *P* < 0.05; ** *P* < 0.01; *** *P* < 0.001 determined by multiple *t* test comparisons (Holm-Šídák, panels **A** and **B**).

Since combination depletion of TNF-α with IL-2, IL-4, or IL-13 eliminates mortality and reduces morbidity in *BALB/c* mice, we studied signaling pathways downstream of their receptors. Qualitative studies in heart, liver, and kidney protein extracts validated phosphorylation of NF-κB pathway component p65 (downstream of TNF-α receptor), STAT5 (downstream of IL-2 receptor), and STAT6 (downstream of IL-4 and IL-13 receptor complex) in Cocktail D–injected *BALB/c* and *BALB/cJ* mice at 15, 30, and 60 minutes but not saline-injected controls (examples, Supplemental Figure 9A). Next, nuclear and cytosolic proteins were extracted from each organ and quality tested for predominant expression of nuclear protein lamin B1 in nuclear extracts (examples, Supplemental Figure 9B) and GAPDH in both fractions (examples, Supplemental Figure 9C). Since IL-2, IL-4, and IL-13 receptors are expressed in the same cells as ZHX2, whereas TNF-α receptors are mostly vascular, Western blot and densitometry quantification of nuclear and cytosolic phosphorylated (p-) STAT5 and p-STAT6 proteins relative to lamin B1, STAT5, and STAT6 were compared in *BALB/c* and *BALB/cJ* mice (Figure 7, A and B; examples, Supplemental Figure 9, D and E). Saline groups did not activate signaling pathways and are not shown. Higher and/or earlier nuclear p-STAT5 expression was noted in all 3 organs in *BALB/cJ* compared with *BALB/c* mice, despite equivalent or lower relative p-STAT5 expression in the cytosolic compartment in most scenarios, especially in the heart and liver (Figure 7A). Overall relative cytosolic p-STAT6 generation was lower in *BALB/cJ* mice at some points, but relative nuclear p-STAT6 expression at those times was equivalent and in some cases higher in *BALB/cJ* compared with *BALB/c* mice (Figure 7B). These data suggest that p-STAT5 and p-STAT6 move into the nucleus more rapidly and earlier in *Zhx2*-deficient *BALB/cJ* mice compared with *BALB/c* mice, causing asynchronous activation of target genes in *BALB/cJ* mice, which could prevent more severe injury and higher mortality in *BALB/c* mice.

### STAT6 pathway– and ZHX-mediated mechanisms in cytokine cocktails induce glomerular injury.

**Figure 8:**
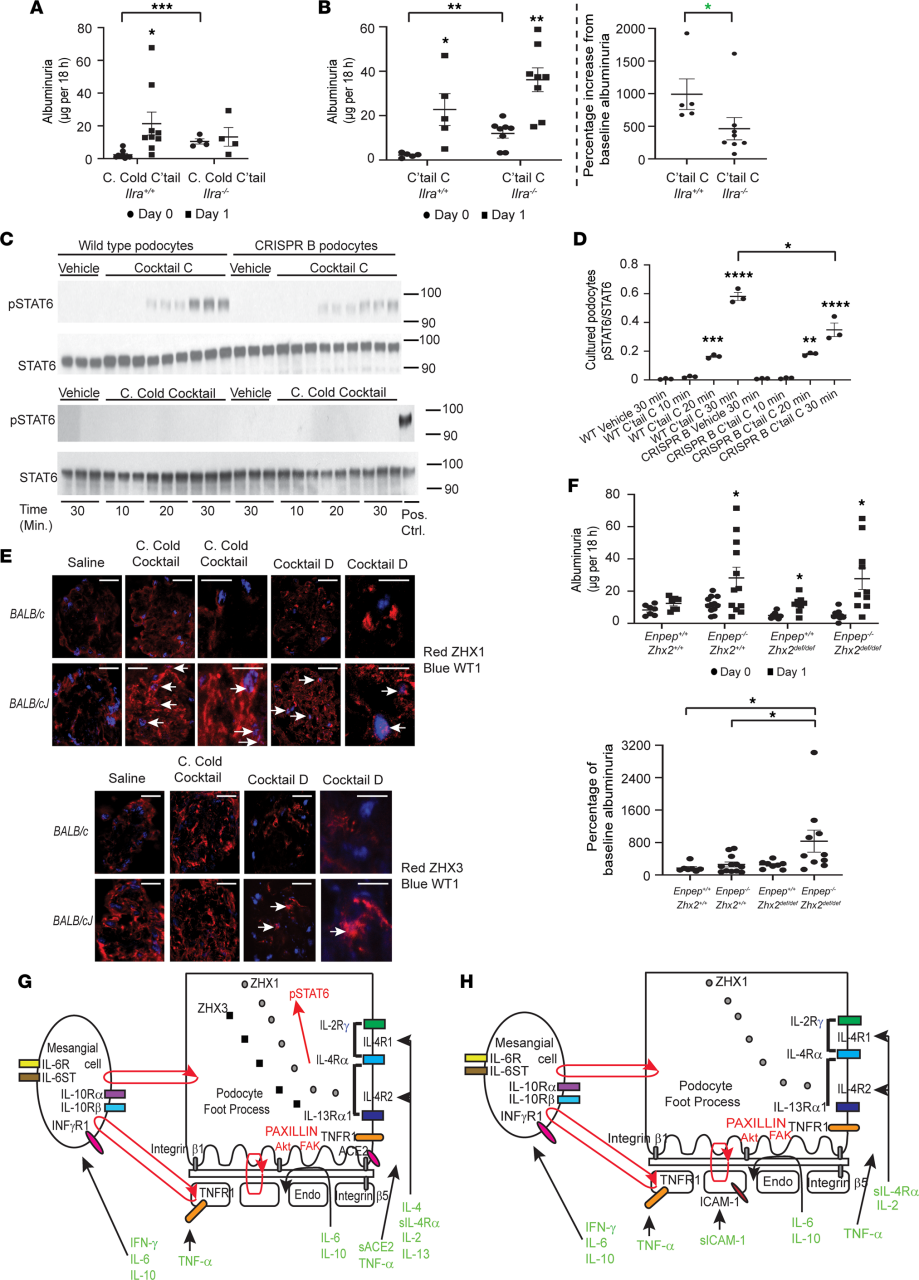
Mechanisms of cytokine storm–related glomerular injury. (**A**) Common Cold (C. Cold) cocktail X/2–induced albuminuria in *Il4ra^–/–^* (*BALB/cJ* background; *n* = 4 mice/group) and control *BALB/cJ* mice (*n* = 9 mice/group). (**B**) Cocktail C X/2–induced albuminuria in *Il4ra^–/–^* (*n* = 8 mice/group) and control *BALB/cJ* mice (*n* = 5 mice/group) (left) and percentage increase in day 1 albuminuria from baseline (right). (**C**) Western blots to assess activation of p-STAT6 signaling in wild-type and *ZHX2* hypomorph (CRISPR B) cultured human podocytes incubated with human counterparts of Cocktail C (upper) or Common Cold cocktail (lower; final concentration X/100,000; *n* = 3 dishes/condition). Positive control for the Common Cold cocktail incubation study was 30-minute Cocktail C incubation in wild-type podocytes. Numbers on right represent kilodaltons. (**D**) Densitometry of Western blots from **C**, upper. (**E**) Confocal images of glomeruli from control saline–, Common Cold cocktail X/2–, or Cocktail D X/2–injected *BALB/c* and *BALB/cJ* mice, showing increased podocyte nuclear presence of ZHX1 (white arrows, upper panel) exclusively in Common Cold cocktail– and Cocktail D–injected *BALB/cJ* mice. Some podocyte nuclei in *BALB/cJ* mice injected with Cocktail D also showed increased ZHX3 (white arrows, lower panel). (**F**) Common Cold cocktail X/5 induced albuminuria (upper) and percentage increase in baseline albuminuria (lower) in *Zhx2^def/def^*, *Enpep^–/–^*, and dual *Zhx2^def/def^* *Enpep^–/–^* mice in mixed background (*n* = 7 to 12 mice/group). def, deficient. (**G**) Schematic for potential binding of COVID-19 cocktail components to specific receptors in glomerular endothelial cells and feedback loops (red) between these cells. (**H**) Schematic for potential binding of Common Cold cocktail components to specific receptors in glomerular endothelial cells and potential feedback loops (red) between these cells. Confocal microscopy scale bars: 10 μm. * *P* < 0.05; ** *P* < 0.01; *** *P* < 0.001; **** *P* < 0.0001, determined by multiple *t* test comparisons (Holm-Šídák, **A**, **B**, **F** top), simple *t* test 2-tail (**A**, **B**, **D**), simple *t* test 1-tail (green, **B**), 1-way Anova (Dunnett **D**; Tukey **F** bottom). Data represent mean ± SEM.

Previously described receptor protein expression patterns in glomeruli and tubules were validated (Supplemental Figure 10, A and B). *Il4ra^–/–^* *BALB/cJ* mice had higher baseline albuminuria than *Il4ra^+/+^* *BALB/cJ* mice (Figure 8, A and B), but did not develop increased albuminuria, after injection of the Common Cold cocktail (Figure 8A), suggesting a central role of IL-4Rα in this model. Both *Il4ra^+/+^* *BALB/cJ* and *Il4ra^–/–^* *BALB/cJ* mice developed higher albuminuria after injection of Cocktail C (Figure 8B, left panel). However, in 1 of 2 studies, the percentage increase in albuminuria from baseline was significantly lower in *Il4ra^–/–^* *BALB/cJ* mice (Figure 8B, right panel), and histological changes were milder in these mice (Supplemental Figure 10, C and D), suggesting a substantial quantitative contribution of IL-4Rα in this model. In vitro, p-STAT6, the major signaling pathway downstream of IL-4Rα, was activated by Cocktail C (contains IL-4) in cultured control and *ZHX2* hypomorph CRISPR B podocytes (Figure 8, C and D) but not by the Common Cold cocktail (no IL-4, Figure 8C). Similar to cytosolic p-STAT6 studies in *BALB/cJ* mouse kidney and liver (Figure 7B), Cocktail C–induced p-STAT6 phosphorylation was significantly lower at 30 minutes in CRISPR B *ZHX2* hypomorph podocytes than wild-type control (Figure 8, C and D). To determine additional podocyte IL-4Rα–related mechanisms active in the common cold model, ZHX protein translocation from the podocyte cell membrane to the nucleus was assessed using confocal imaging in *BALB/c* and *BALB/cJ* mouse glomeruli 1 day after injecting the Common Cold cocktail (Figure 8E). Uniform increased nuclear ZHX1 was noted in *BALB/cJ* but not *BALB/c* mice. Similar changes in podocyte ZHX1, and increased ZHX3, were noted in some podocyte nuclei in Cocktail D–injected *BALB/cJ* mice. In normal in vivo podocytes, ZHX2-ZHX1 heterodimers bind the cytoplasmic aspect of transmembrane protein APA (27), and increased severity of experimental glomerular injury in *Zhx2*-deficient and glutamyl aminopeptidase-knockout (*Enpep^–/–^*) mice (27) led us to postulate putative alternative, lower affinity binding partners for ZHX proteins in both conditions. Since both cocktails induced translocation of cell membrane ZHX1 into podocyte nuclei selectively in *BALB/cJ* mice, we considered whether IL-4Rα was an alternative binding partner for ZHX proteins in *Zhx2* deficiency. We co-immunoprecipitated IL-4Rα and ZHX1 selectively from *ZHX2*-deficient podocytes (Supplemental Figure 10E). To further increase ZHX1 binding to alternative partners and test if that affected glomerular disease severity, *Zhx2*-deficient, *Enpep^–/–^*, or dual *Zhx2*-deficient *Enpep^–/–^* mice were injected with the strain-specific Common Cold cocktail threshold nephritogenic dose X/5 (Figure 8F). In addition to *Zhx2*-deficient mice, *Enpep^–/–^* mice had increased albuminuria at comparable levels, supporting the alternative binding partner scenario. The highest albuminuria was noted in dual *Zhx2*-deficient *Enpep^–/–^* mice. Injecting dual *Zhx2*-deficient *Enpep^–/–^* mice with the threshold nephritogenic dose for *BALB/cJ* mice (Common Cold cocktail dose X/2) resulted in several-fold higher albuminuria (Supplemental Figure 10F), thereby validating the pathogenic importance of ZHX-mediating mechanisms in cytokine-induced disease. Therefore, both ZHX- and p-STAT6–mediated mechanisms related to podocyte IL-4Rα are active in COVID-19 models (Figure 8G) and ZHX mechanisms only in the common cold model (Figure 8H).

## Discussion

SARS-CoV-2 infection of the respiratory tract elicits a prominent immune response, and in some cases, involves other organs by direct infection (6). The magnitude of the extrapulmonary involvement is often out of proportion to direct infection, suggesting the innate and adaptive immune response to the primary infection may have a significant pathogenic role (4, 6). Whereas the immune response could include immune cells, immunoglobulins, complement, cytokines, chemokines, acute-phase reactants, and other components, this study focuses on the multisystem pathogenic effects of the extensive cytokine storm documented early in the pandemic (1). While not all components of human cytokine storms are included in the cocktails, these cocktails are clearly sufficient to produce the extrapulmonary manifestations of viral infections studied.

Two viral cytokine storm models (rhinovirus common cold and SARS-CoV-2/COVID-19) with partially overlapping components were developed and tested under similar experimental conditions to study synergistic rather than individual effects of cytokines. Common cold viruses cause local upper respiratory tract infection, and the accompanying constitutional symptoms are commonly attributed to the associated mild cytokine storm. Relapse of rare diseases like MCD and FSGS after a common cold is a well-defined clinical phenomenon (26) that represents an uncommon nonrespiratory organ manifestation of this illness. The use of glomerular disease to study mild cytokine storms allowed us to compare the effects of 2 common viral infections on rare (e.g., relapse of MCD by common cold) and common (COVID-19–induced proteinuria) clinical scenarios in the absence of other end organ damage. Whereas the Common Cold cocktail induced albuminuria selectively in the *Zhx2* hypomorph state, simply substituting ACE2 for ICAM-1 in Common Cold cocktails (COVID-19 Cocktail A) induced albuminuria in *Zhx2^+/+^* mice. The more potent transition from Common Cold to COVID-19 cocktails was the inclusion of allergy pathway cytokines IL-4 in Cocktail C and IL-13 and IL-4 in Cocktails B and D. Receptors for these 2 cytokines function as a complex in podocytes, and both cytokine storm models induced lesser albuminuria in *Il4ra^–/–^ Zhx2^hypo/hypo^* mice. Some cytokine components of these viral cytokine storm cocktails are also noted in bacterial sepsis–related cytokine release. However, high circulating levels of allergy pathway cytokines (IL-13, IL-4), a prominent feature in severe SARS-CoV-2 infection (1), are uncommon in most bacterial infections. Also, IFN release (IFN-γ in our studies) is also more prominent in viral infections and accounts for the high dose of IFN-γ in our cocktails. Mice required μM amounts (corresponds to nanomolar or high picomolar plasma levels) and rats nM amounts (corresponds to picomolar levels) of the cytokine cocktail to induce disease. Patients with COVID-19 in intensive care units have low to high picomolar levels of most cytokines (1) in our COVID-19 cocktails.

At higher COVID-19 cocktail doses, systemic effects beyond glomerular injury, including acute myocarditis, pericarditis, liver and kidney injury, and substantial acute all-cause mortality, were noted. Since the cytokine storm origin was extrinsic to these organs, only mild to moderate inflammation, as also often noted in SARS-CoV-2–infected patients, was present. Compared with *BALB/c* mice, *Zhx2^hypo/hypo^* *BALB/cJ* mice developed less severe heart, liver, and kidney injury and lower mortality, whereas the extent of glomerular injury was similar. The disparity between glomerular and other forms of injury is possibly related to the predominantly cell membrane localization of ZHX proteins in podocytes (27, 40, 41), and largely nuclear expression in the heart (42), liver (43), and kidney tubular cells (27), though the use of low-confidence and polyreactive antibodies to stain human tissue in a key resource (42) needs to be resolved. The protection from mortality and multiorgan toxicity offered by the *Zhx2^hypo/hypo^* state could have therapeutic implications. Most of the therapeutic principles explored in this study are based on more readily translatable cytokine depletion. The depleting antibodies were administered 1 hour after injection of the cytokine cocktail, which is sufficient time to initiate multi-pathway injury, since all mice injected with high-dose Cocktail D were equally sick at 6 hours. The improvement, or its lack, at 24 hours was reflective of the therapeutic efficacy of the depletion regimen. The most effective regimens for severe cytokine storms in *BALB/c* mice with no mortality and normal/near normal biomarker levels were combination depletion of TNF-α with IL-2, IL-4, or IL-13. Monotherapy for IL-2, TNF-α, IL-4, and IL-13 depletion also eliminated mortality, and overall activity was improved. However, either biomarker levels tended to be higher than the combination groups, or signs of distress, e.g., standing hair, persisted. Groups with monotherapy for depletion of IL-6 or IL-10 fared worse than other groups. By contrast, IL-6 depletion was highly therapeutic in *BALB/cJ* mice, second only to TNF-α/IL-2 combination depletion. Monotherapy for IFN-γ depletion was also effective in *BALB/cJ* mice, whereas monotherapy for depletion of TNF-α or IL-10 or IL-4 was counterproductive, even though the combination was effective. The dependence of IL-6 depletion effects on *Zhx2* expression may explain some of the heterogeneity of early clinical trials in patients with COVID-19 (44–47), since IL-6 depletion is still used in some COVID-19 clinical settings. In the intermediate-dose Cocktail D 1.8× model in *BALB/c* mice, select single-cytokine depletion was effective. In mild cytokine storm models (1× or lower dose), monotherapy for TNF-α, IL-6, or IL-4Rα depletion was effective in reducing common cold–induced albuminuria, suggesting a potential therapeutic approach to prevent MCD or FSGS relapse. In mild Cocktail C models, anti–TNF-α antibody and select anti–TNF-α antibody–based combinations were effective in reducing albuminuria.

These studies showcase the concept of synergy between different cytokines, compartments, and signaling pathways in disease pathogenesis. Cytokine depletion regimens described in this study also affect this synergy. In the glomerulus, cytokine receptor expression is distributed between podocytes and endothelial and mesangial cells (Figure 8G), which suggests a fundamental role of altered crosstalk between these cells in disease pathogenesis. In the kidney tubulointerstitial compartment, TNFR1 is expressed in vascular endothelium, all other receptors and ACE2 in the proximal tubule, and many receptors in distal tubules and the collecting duct (Supplemental Figure 11A). This distribution supports cytokine storm–related direct tubular injury in AKI. Moreover, the pattern of tubular injury in both 1.8× and 3× injury models in *BALB/c* mice is discontinuous, suggesting predominantly toxic cytokine mediated, rather than hemodynamic injury. In the myocardium, major receptor expression in coronary vascular endothelium and smooth muscle cells, and lower expression in fibroblasts and cardiomyocytes, point toward a vascular pathogenesis of cytokine storm–related acute myocardial injury (Supplemental Figure 11B). In the liver, receptors are distributed evenly between various cell types, suggesting multisite toxicity of the cytokine storm (Supplemental Figure 11C).

Asynchronous activation of nuclear targets of p-STAT5 and p-STAT6 in *BALB/cJ* compared with *BALB/c* mice could account for some aspects of reduced injury and mortality from COVID-19 cocktail injection in *BALB/cJ* mice. In heart, liver, and kidney, nuclear p-STAT5 expression was mostly higher in *BALB/cJ* mice in the time studied, and increased sooner, despite mostly comparable or even lower levels in the cytosol. By contrast, nuclear p-STAT6 was equivalent between the strains, and sometimes higher in *BALB/cJ* mice (e.g., liver), despite significantly lower (Figure 7) cytosolic p-STAT6 in many *BALB/cJ* mouse samples. These data suggest that the *Zhx2* hypomorph state is associated with early and rapid transit of p-STAT5 and p-STAT6 from cytosol into the nucleus and with reduced p-STAT6 generation upon cytokine cocktail activation. Future studies will explore the potential effects of transcriptional factor *ZHX2* on nuclear pore protein gene expression, since mutations in one of these genes have been noted in a family with FSGS collapsing variant (48).

At least 2 of likely numerous pathways active in podocytes during cytokine storms were defined. Migration of peripheral ZHX1 into podocyte nuclei, previously shown to increase albuminuria by increasing expression of MCD mediators like angiopoietin-like 4 (ANGPTL4) (27, 49), was also noted in *Zhx2^hypo/hypo^* mice injected with both cytokine cocktails at low doses. Deficiency of APA, the normal ZHX2-ZHX1 transmembrane anchor protein (27), and ZHX2 in podocytes appears to promote binding of ZHX1 to IL-4Rα. This alternative binding state promoted cytokine cocktail–induced albuminuria in *Zhx2^hypo/hypo^* and *Enpep^–/–^* mice and was more severe in dual *Enpep^–/–^ Zhx2^hypo/hypo^* mice. Migration of ZHX3, an *ANGPTL4* repressor, from the slit diaphragm into the podocyte nucleus by COVID-19 cocktails parallels similar observations in human and experimental FSGS (27). ZHX protein translocations from cell membrane to nucleus cannot be studied in cultured podocytes, since nearly half of ZHX proteins are expressed in the nucleus at baseline in vitro (41). p-STAT6 signaling, the other major mechanism studied in podocytes, was activated downstream of IL-4Rα by COVID-19 cocktails in the *ZHX2^+/+^* podocytes and reduced in *ZHX2^hypo/hypo^* cells. In addition, the pathogenic effects of circulating sACE2 in COVID-19 cocktails could be mediated via interaction with integrins (50). The increase in plasma sIL-4Rα levels in COVID-19 patients with proteinuria suggests that this pathway is active in this subset of patients.

This study also offers insight into disease mechanisms behind relapse of human primary glomerular diseases related to a common cold. It steers away from a search for single causal proteins triggered by a 5-decade-old hypothesis (51) toward a clinically and mechanistically relevant protein complex involved in disease relapse. Transient albuminuria is noted, since these mice do not have additional pathway defects, such as abnormal sialylation (49), or additional disease-causing gene variants (52, 53). The common cold model, however, does provide the last missing piece of the MCD puzzle. A previous study (49) describes a sialylation defect in highly upregulated podocyte *Angptl4* as being a critical determinant of initiation of proteinuria in MCD. A follow-up study (27) showed mechanisms of podocyte *Angptl4* upregulation by ZHX1 and how a *Zhx2* hypomorph state predisposes to the cell membrane to nuclear migration of ZHX1. The current study shows that a common cold cytokine storm triggers this nuclear migration of ZHX1 bound to podocyte IL-4Rα in the Zhx2 hypomorph state.

This study also provides a genomic basis for altered constitutive podocyte ZHX2 expression in MCD and FSGS. The insertion at Chr8: 122,533,694 was shared among patients with MCD and FSGS, and replication in a cultured podocyte cell line induced *ZHX2* downregulation, adding to rapidly accumulating evidence (27) of *ZHX2* as an important disease modifier gene in primary glomerular diseases. Whether the 3 other shared InDels increase or reduce *ZHX2* expression in COVID-19 and collapsing variant FSGS patients will require CRISPR/Cas9 replication in cultured podocytes. However, published literature supports a role of high, not low, podocyte *Zhx2* expression in experimental collapsing glomerulopathy (27). Adriamycin injection studies in 3 lines of podocyte-specific *Zhx2*-overexpressing transgenic rats show development of collapsing glomerulopathy beyond a certain threshold of *Zhx2* overexpression (27). In our limited studies, there was no evidence that *APOL1* gene variants in collapsing glomerulopathy extended beyond the African American patient SF19, who also had 3 single InDels in the *HAS2–ZHX2* intergenic region. The *HAS2–ZHX2* interval appears to be a site for evolutionary recombination, since the prostaglandin transporter gene *Slc22a22* (size 233,858 bp in mice) is 8 times larger in mice compared with rats (size 29,686 bp) because of the absence of a large intron and noncoding exon. In larger animals, and in humans, this gene is inactive. In addition, *ZHX1* is present only a few genes downstream of *ZHX2* on chromosome 8. Half of patients with FSGS with known disease-causing variants in *INF2*, *NPHS2*, and *WT1* (52, 53) had additional insertions and/or deletions in this region. These InDels may be acting in concert with single-gene causes of FSGS to induce disease, since many disease-causing variants identified through exon sequencing are present in seemingly healthy individuals in genome databases like the 1000 Genomes Project (54).

Since Omicron-lineage SARS-CoV-2 strains cause pulmonary disease less frequently compared with the original stain, it is possible that the cytokine storm is also less severe with these evolving variants due to reduced respiratory tract involvement. Nevertheless, therapeutic principles developed here for critically ill intensive care unit patients, most of whom have significant pulmonary involvement, will remain unchanged. The most important lesson learned is the use of combination cytokine depletion in the future to treat severe cytokine storms, rather than single-cytokine depletion. Even though we have tried to adjust for multiple comparisons, there is still a possibility of type I errors due to the large number of tests performed. COVID-19 cocktails could be extended by longer term infusion to design models for post acute sequelae of SARS-CoV-2 infection (called PASC or long COVID). Some rare but significant side effects of SARS-CoV-2 mRNA vaccines, such as myocarditis and pericarditis (55), may result from an exaggerated cytokine response following vaccination, in which case cytokine depletion strategies from the intermediate- or high-dose BALB/c models can be considered. Finally, therapeutic principles and strategies discussed in this paper could be used for other cytokine storms with an overlapping spectrum.

## Methods

### COVID-19 and common cold cytokine cocktails and related animal studies.

Methods for dynabead-assisted mouse glomerular isolation, rat glomerular isolation by sieving, histological section tissue preservation, timed 18-hour urine collection in metabolic cages in the absence of food, assessment of albuminuria and proteinuria, real-time PCR, confocal imaging, electron microscopy and sample processing, histology for light microscopy, Western blot, co-immunoprecipitation, and *APOL1* genotyping were previously described (27, 40, 41, 48, 56–58). The following were assayed using commercially available kits using serum samples: mouse ALT (BioVision: K752-100), mouse cTPI3 (Novus Biologicals: NBP3-00456), mouse CK (Abcam: ab155901), and human IL-4Rα ELISA (Abcam: ab46022). Antibodies against ZHX1, ZHX2, and ZHX3 were previously described (27, 40, 41). Real-time PCR FAM-MGB probes for cytokine receptors, ACE2, STAT5, STAT6, and NF-κB–p-65 were purchased from Thermo Fisher Scientific. Serum and urine creatinine were assayed by mass spectrometry. Details of these methods are included in the Supplemental Methods.

All cytokines, soluble receptors, and antibodies were injected intravenously in rodents and are listed in Supplemental Table 3. Antibodies used for depletion studies were characterized by Western blot using the corresponding recombinant protein (Supplemental Figure 10G). Each dose of cytokine cocktail was dissolved in a final volume of 100 μL of sterile 0.9% saline. *BALB/cJ* (The Jackson Laboratory) and *BALB/c* (Envigo) mice were purchased at age 8 weeks, then acclimatized for 2 weeks, and baseline 18-hour urine collection and tail blood sampling were conducted. An extra baseline urine collection was conducted for *BALB/cJ* mice. Most in vivo studies were conducted between age 10 and 15 weeks. *Enpep^–/–^* *Zhx2^def/def^* in mixed background were obtained by interbreeding the F_2_ cross between *Enpep^–/–^* (27) and *Zhx2*-deficient *BALB/cJ* mice. The nephritogenic dose spectrum of cytokine cocktails was established for *BALB/cJ*, *BALB/c*, *Il4r^–/–^* (The Jackson Laboratory), *Zhx2^fl/fl^ NPHS2 promoter^cre/cre^*, *Enpep^–/–^*, and *Enpep^–/–^* *Zhx2^def/def^* mice. During mouse cytokine studies using threshold nephritogenic doses (*BALB/cJ*, *BALB/c*, *Il4r^–/–^* in *BALB/cJ* background, X/2; *Zhx2^fl/fl^ NPHS2 promoter^cre/cre^*, X/15; *Enpep^–/–^* *Zhx2^def/def^* studies, X/5), 100 μL of 0.9% saline was given intraperitoneally immediately after the intravenous cytokine cocktail dose to maintain intravascular hydration. Two additional intraperitoneal injections of 100 μL of 0.9% saline were given at 6 and 23 hours in the intermediate- and high-dose cocktail models. During cytokine depletion studies, different groups of mice received 50 μg of control IgG or the respective antibody or antibody combination intravenously 1 hour after the administration of the mouse cytokine cocktail. During Buffalo Mna rat (obtained via a Material Transfer Agreement with Kyoto University, Kyoto, Japan) cytokine studies using threshold nephritogenic dose (X/50), male rats (*n* = 7) with baseline proteinuria between 35 and 63 mg at 18 hours were injected intravenously with X/50 cytokine cocktail dose dissolved in 100 μL sterile 0.9% saline, followed immediately by 1 mL of 0.9% saline intraperitoneally to maintain intravascular hydration. Timed urine collections (18 hours) were conducted on days 1, 3, 5, and 7, and the peak increase in proteinuria was noted for each animal.

### Sources of human genomic DNA and human kidney biopsies.

Genomic DNA samples from 36 patients with nephrotic syndrome, 33 control subjects, and 16 patients with diabetic nephropathy (Supplemental Tables 1 and 2) were obtained from the following sources: a) Immortalized monocytes from plasma of patients with nephrotic syndrome at the University of Alabama at Birmingham. b) Instituto Nacional de Cardiología in Mexico City for archived kidney biopsies from patients with glomerular diseases or preimplantation kidney biopsies from healthy living related kidney donors (27). c) Archived kidney biopsies from Hospital Nacional Alberto Sabogal Essalud, Lima, Peru (gift from Julia Sumire-Umeres). d) Archived human DNA of previously published FSGS patient cohort (52, 53) from the Duke Molecular Physiology Institute with known mutations in podocyte-expressed genes. e) Coriell Cell Repositories, which archive DNA from the 1000 Genomes Project and the HAPMAP Project.

For analytical comparisons between cases and controls, the 1000 Genomes Project phase 3 Ensembl v84 (2,504 participants) was included as an additional control. Methods of Agilent custom capture and high-throughput Illumina sequencing are included in the Supplemental Methods.

### Genome editing in cultured human podocytes using CRISPR/Cas9.

The basic methodology for CRISPR/Cas9 was previously published (59). A single cell–derived clone of cells was generated from an established early-passage immortalized human podocyte cell line (gift from Moin Saleem, University of Bristol, Bristol, United Kingdom) (60) and used for genome-editing studies. The oligonucleotides and primers used are listed in Supplemental Table 4. Specific methodological details are provided in the Supplemental Methods.

### STAT5, STAT6, and NF-κB pathway studies in animal models.

See Supplemental Methods.

### In vitro STAT6 signaling studies.

See Supplemental Methods.

### Human plasma from COVID-19 and control patients for IL-4Rα assay.

See Supplemental Methods.

### Statistics.

Multiple comparisons with a single group were done by 1-way ANOVA, using Dunnett’s multiple comparisons test (majority of tests) or by controlling the FDR using the method of Benjamini, Krieger, and Yekutieli (*q* = 0.05). When comparing every mean with every other mean, the Tukey test was used. When using multiple *t* test comparisons, Holm-Šídák correction (majority of tests) or FDR method was used. Simple unpaired 1- or 2-tailed *t* test was used to compare 2 groups. For statistical analysis and graphical illustrations, GraphPad Prism 9.5.1 was used.

### Study approval.

Human DNA samples for sequencing were covered by the following institutional study approvals: a) University of Alabama at Birmingham, IRB-approved protocol X080813001 for collecting DNA and blood and urine samples. b) Instituto Nacional de Cardiología in Mexico City IRB-approved studies CONACYT 34751M, CONACYT 11-05, and DPAGA-UNAM IN-201902 that included archived kidney biopsies from patients with glomerular diseases or preimplantation protocol kidney biopsies from healthy living related kidney donors (27). c) Archived kidney biopsies, IRB exempt, from Hospital Nacional Alberto Sabogal Essalud, Lima, Peru. Also, deidentified COVID-19 patient plasma was obtained from IRB-approved studies from Rush University COVID-19 Registry and Biorepository. All animal studies were approved by the IACUC at Rush University or the University of Alabama at Birmingham. All animals received humane treatment per protocol.

### Data availability.

Data sets are deposited at the National Center for Biotechnology Information BioProject repository under BioProject ID PRJNA940110.

## Author contributions

MDNA conducted common cold cytokine studies and generated and characterized CRISPR/Cas9 constructs and cell lines. RD, JK, and EMJ conducted glomerular COVID-19 cytokine studies, primary and secondary genomic screening and analysis with Sumant SC, and mass spectrometry creatinine assays. HDB and SG conducted select animal studies. MC and DKC conducted custom capture studies and high-throughput sequencing. RAG assisted with study design and analysis of genomic screens and conducted *APOL1* genotyping. Sunveer SC and Sunjeet SC conducted whole-organ and nuclear and cytosolic fraction Western blot signaling studies. CAC conducted histological analysis of rodent tissue sections. CM conducted assays for multiorgan effect studies related to COVID-19 cocktails. LCC developed logistics and supervised the execution of mouse COVID-19 cytokine studies, injected all mice for the COVID-19 models, conducted all confocal imaging, and reformatted manuscript figures to journal specifications. Sumant SC envisioned the overall concept of the study; envisioned, developed, and designed common cold and COVID-19 cytokine cocktails; designed all cytokine and knockout mouse studies; designed all multiorgan toxicity and therapeutics studies; conducted primary genomic analysis using QIAGEN and IGV software; conducted densitometry and some Western blot studies; and wrote the manuscript.

## Supplementary Material

Supplemental data

## Figures and Tables

**Table 1:**
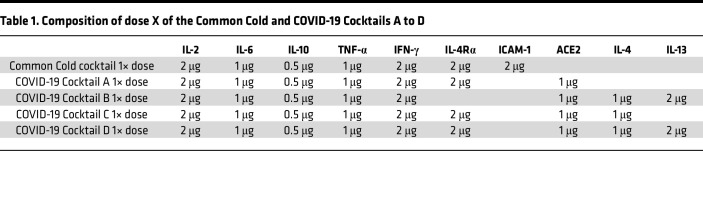
Composition of dose X of the Common Cold and COVID-19 Cocktails A to D

**Table 2:**
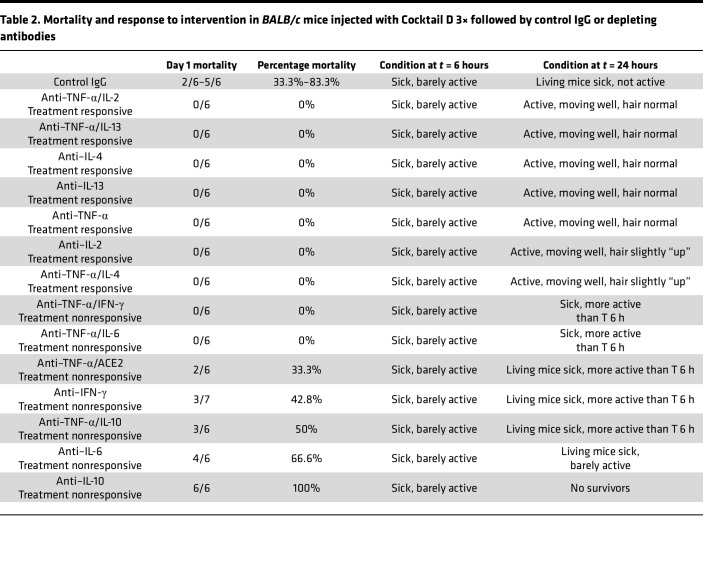
Mortality and response to intervention in *BALB/c* mice injected with Cocktail D 3× followed by control IgG or depleting antibodies

**Table 3:**
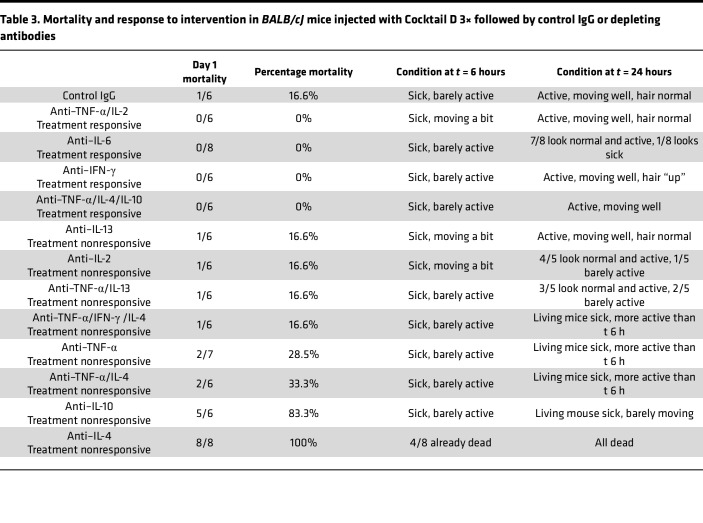
Mortality and response to intervention in *BALB/cJ* mice injected with Cocktail D 3× followed by control IgG or depleting antibodies

**Table 4:**
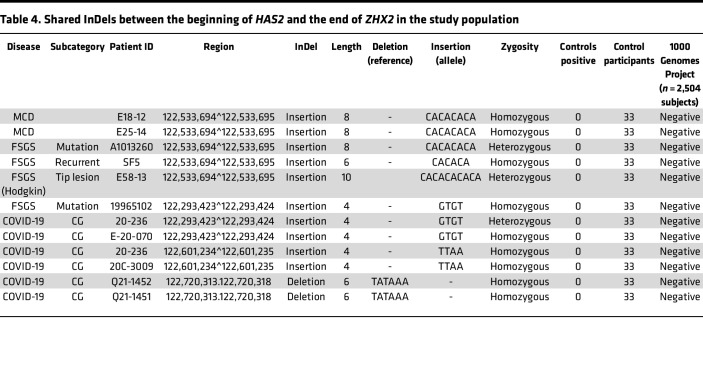
Shared InDels between the beginning of *HAS2* and the end of *ZHX2* in the study population

## Version 1 04/11/2023

In-Press Preview

## Version 2 05/22/2023

Electronic publication

## Version 3 05/24/2023

Rasheed Gbadegesin's name was corrected
