# Supplementary material for: Cytokine storm–based mechanisms for extrapulmonary manifestations of SARS-CoV-2 infection
Source: JCI Insight. 2023 May 22;8(10):e166012. doi: 10.1172/jci.insight.166012 (PMC10322692; doi:10.1172/jci.insight.166012)
Supplement: Supplemental data [file jciinsight-8-166012-s013.pdf]

SUPPLEMENTARY INFORMATION

Table of Contents

Table of Contents: Page 1

Figures: Pages 2 - 22

Tables: Pages 23 - 26

Methods: Pages 27 - 32

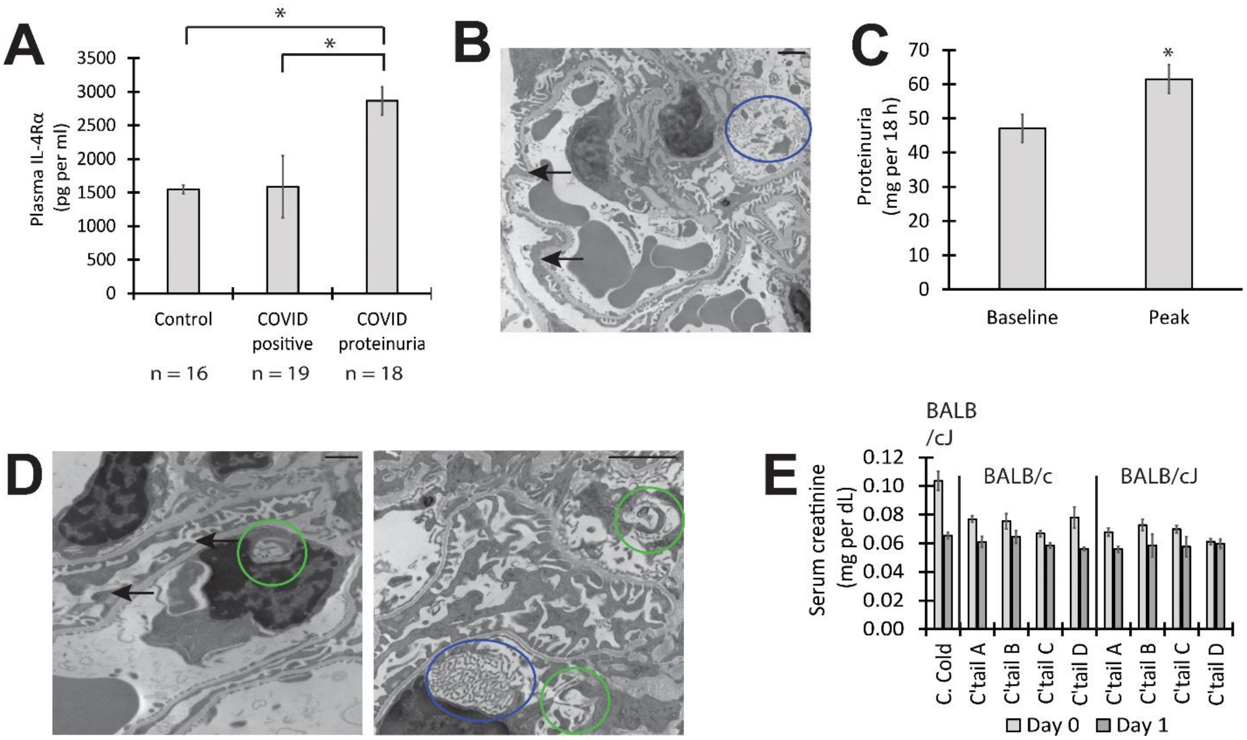

Supplementary Figure 1: Data represent mean ± SEM. (**A**) Plasma IL-4Rα levels assessed by ELISA in general COVID-19 patients, age, sex and race matched healthy controls, and COVID-19 patients with proteinuria. Number of patient samples assayed is shown below. (**B**) Electron microscopy of *BALB/cJ* mouse glomeruli 24 hours after injecting Common Cold cocktail dose X/2. Areas of focal foot process effacement (black arrows) and endothelial hypertrophy (blue circles) were noted. (**C**) Peak change in proteinuria from baseline in Buffalo Mna rats (n = 7 male rats; age 3 months) up to 7 days after injection of the rat Common Cold cocktail at threshold nephritogenic dose X/50. (**D**) Electron microscopy images of *BALB/cJ* mouse glomeruli on Day 1 after injection of Cocktail D dose X/2. Areas of focal foot process effacement (black arrows), endothelial vacuolation (green circles), and endothelial hypertrophy (blue circles) were noted. (**E**) Serum creatinine, assayed by Mass Spectrometry, is not increased in the Common Cold cocktail dose X/2 (n = 6 *BALB/cJ* mice) and COVID cytokine cocktail dose X/2 models (*BALB/c* and *BALB/cJ* mice; n = 5 - 6 mice per group). Scale bars 0.5 µm. * P<0.05; determined by one-way Anova (Tukey, panel A), simple two-way *t* test (panel C).

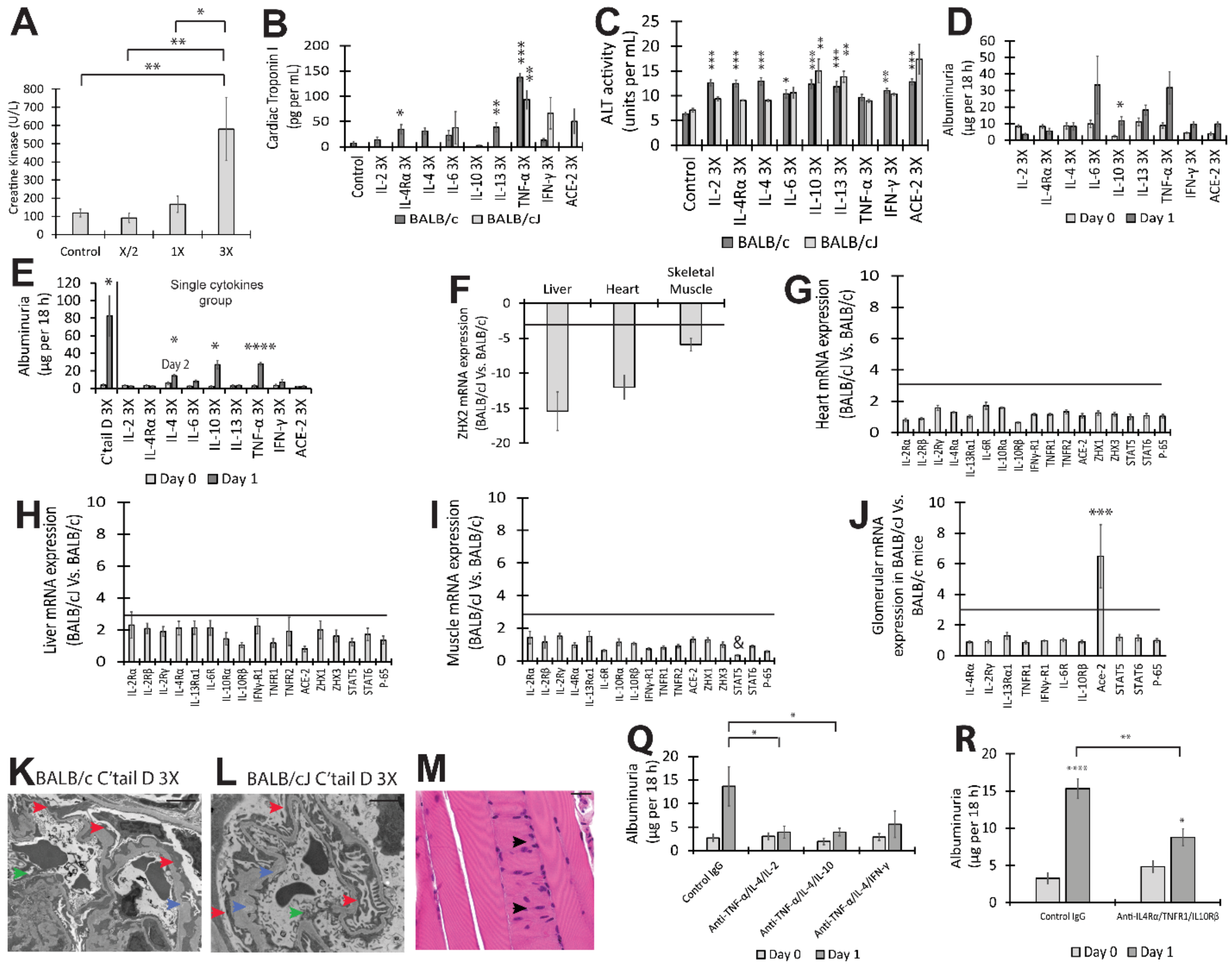

N

Mouse Strain	Myocytolysis	Fibril Disruption	Hypereosinophilia	Inflammation	Pericarditis
BALB/c	Yes	Present	Focal	Mild	Yes
BALB/cJ	Yes	Minimal	Focal	Moderate	No

O

Mouse Strain	Hepatocellular Injury	Inflammation	Degenerative Changes	Regenerative Changes	Peri-central Vein Injury
BALB/c	Marked	Minimal, Prominent Kupfer cells	Frequent	Frequent	No
BALB/cJ	Extensive	Moderate	Frequent	Frequent	Yes

P

Mouse Strain	Proximal Tubular Changes			Distal Tubular Changes	
	Vacuolation	Brush Border	Degeneration	Desquamation	Foam Cells
BALB/c	Marked	Disrupted	Multifocal	Yes	Occasional
BALB/cJ	Frequent	Disrupted	Multifocal	Yes	Frequent

Supplementary Figure 2: Data represent mean ± SEM. (**A**) Plasma creatine kinase, a marker of skeletal muscle injury, in *BALB/cJ* mice (n = 4 - 5 mice per group) 24 hours after injection of Cocktail D at different doses. (**B**) Serum Cardiac Troponin I level data derived from Fig. 2A, plotted again for higher resolution of lesser increase in levels among some single cytokine injected groups. (**C**) Serum ALT level data derived from Fig. 2B, plotted again for higher resolution of lesser increase in levels among some single cytokine injected groups. (**D**) 18-hour albuminuria in *BALB/c* mice injected with single cytokine dose 3X on Day 1, corresponding to Figs. 2A-C (n = 5 mice per group). It is possible that some of these values could be higher on Day 2, especially IL-4, IL-13, corresponding to Figure 1K. Given their high mortality after Cocktail D 3X, metabolic cage housing for timed urine collection is not feasible in *BALB/c* mice. (**E**) 18-hour albuminuria in *BALB/cJ* mice injected with Cocktail D 3X or single cytokines dose 3X, corresponding to Figs. 2A-C (n = 4 - 5 mice per group). (**F**) Fold-difference in heart and skeletal muscle *Zhx2* mRNA expression in *BALB/cJ* compared to *BALB/c* mice assessed by real time PCR (n = 6 templates per group). Lower levels of Zhx2 mRNA expression in the liver were previously published and serve as a positive control for this phenomenon. Three-fold difference was taken as significant. (**G**) Real time PCR comparison of expression of cytokine receptors, *Ace2*, *Zhx1, Zhx3*, and signaling pathway proteins STAT5, STAT6 and P-65 (NFκB) between *BALB/cJ* and *BALB/c* mouse heart (n = 6 templates per group). Three-fold difference was taken as significant. (**H**) Real time PCR comparison of expression of cytokine receptors, *Ace2*, *Zhx1, Zhx3*, and signaling pathway proteins STAT5, STAT6 and P-65 (NFκB) between BALB/cJ and BALB/c mouse liver (n = 6 templates per group). Three-fold difference was taken as significant. (**I**) Real time PCR comparison of expression of cytokine receptors, *Ace2*, *Zhx1, Zhx3*, and signaling pathway proteins STAT5, STAT6 and P-65 (NFκB) between *BALB/cJ* and *BALB/c* mouse skeletal muscle (n = 6 templates per group). Three-fold difference was taken as significant. & 3.06 ± 0.40 fold higher expression of STAT5 in *BALB/c* mice. (**J**) Real time PCR comparison of expression of cytokine receptors, *Ace2*, and signaling pathway proteins STAT5,

STAT6 and P-65 (NFκB) between *BALB/cJ* and *BALB/c* mouse glomeruli (n = 6 templates per group). Three-fold difference was taken as significant. Other IL-2R chains are not expressed in mouse glomeruli, and *Zhx1* and *Zhx3* are previously published. (**K**) Electron microscopy of BALB/c mouse kidney glomeruli 24 hours after injection Cocktail D dose 3X. Extensive foot processes effacement (red arrows), endothelial hypertrophy (green arrows) and glomerular basement membrane (GBM) remodeling (blue arrows) were present. (**L**) Electron microscopy of BALB/cJ mouse kidney glomeruli 24 hours after injection Cocktail D dose 3X. Multifocal foot processes effacement (red arrows), endothelial hypertrophy (green arrows) and glomerular basement membrane (GBM) remodeling (blue arrows) were present. (**M**) Hematoxylin and Eosin stained skeletal muscle from BALB/cJ mice 24 hours after injection of Cocktail D dose 3X. Focal inflammation (black arrows) was noted in some sections. (**N**) Morphometric analysis and comparison of histological changes in the heart in Cocktail D 3X injected *BALB/c* and *BALB/cJ* mice. (**O**) Morphometric analysis and comparison of histological changes in the liver in Cocktail D 3X injected *BALB/c* and *BALB/cJ* mice. (**P**) Morphometric analysis and comparison of histological changes in the kidney in Cocktail D 3X injected *BALB/c* and *BALB/cJ* mice. (**Q**) Albuminuria after induction of the Cocktail D model in *BALB/cJ* mice (n = 5 - 6 mice per group; dose X/2), followed by Control IgG or combinations of depleting antibodies one hour after model induction. (**R**) Albuminuria after induction of Cocktail C in *BALB/c* mice (n = 5 - 6 mice per group; dose X/2), followed by receptor blockage using antibodies against IL-4Rα, TNFR1 and IL-10Rβ, or control IgG. Scale bars (K) 0.5 μm, (l) 0.5 μm, (m) 20 μm. * P<0.05; ** P<0.01; *** P<0.001, **** P<0.0001, determined by one way Anova (Tukey, panel A; Dunnett, panel Q); multiple t test comparison (Holm-Sidak, panels D, E, R), simple two-way t test (panels J, R).

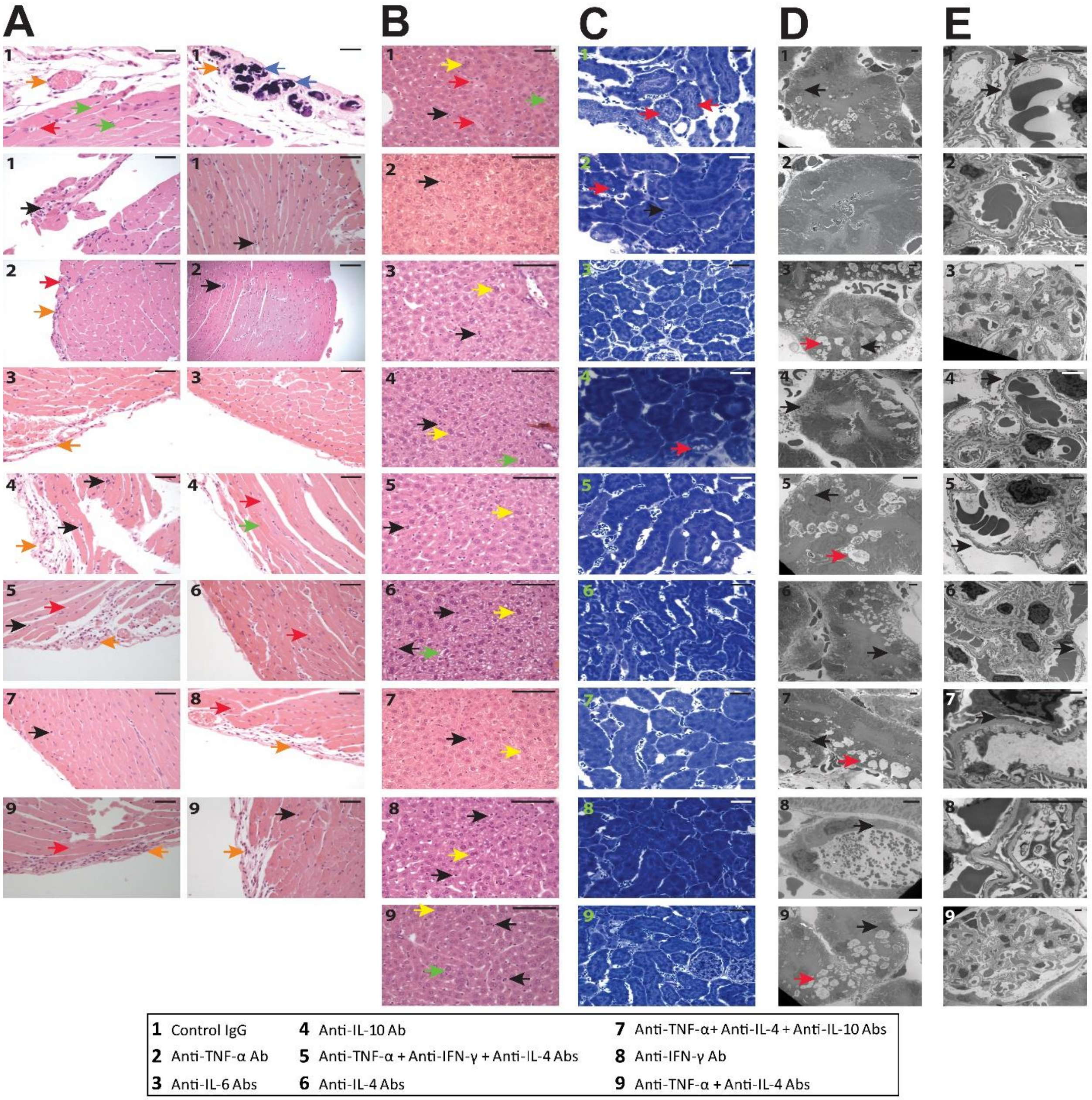

F

Cocktail D 1.8X *BALB/c* Group	Myocytolysis	Hypereosinophilia	Inflammation	Pericarditis
Control IgG	Yes	Yes	Focally dense, perivascular	Focal intense, congestion, microcalcifications
Anti-TNF-α Ab	Yes	No	Focal	Mild to Moderate
Anti-IL-6 Abs	No	No	No	Minimal
Anti-IL-10 Ab	Focal	Yes	Scattered	Focal mild, edema
Anti-TNF-α + Anti-IFN-γ + Anti-IL-4 Abs	Focal injury	No	Focal	Focal mild to moderate
Anti-IL-4 Abs	Very focal	No	No	No
Anti-TNF-α+ Anti-IL-4 + Anti-IL-10 Abs	No	No	Minimal	No
Anti-IFN-γ Ab	Yes	No	No	Moderate
Anti-TNF-α + Anti-IL-4 Abs	Focal	No	Scattered	Mild to Moderate

G

Cocktail D 1.8X *BALB/c* Group	Hepatocellular Injury	Inflammation	Degenerative Changes	Regenerative Changes	Peri-central Vein Injury
Control IgG	Marked	Diffuse and Focal	Yes	Yes	No
Anti-TNF-α Ab	No	Focal	Focal	Frequent binucleated hepatocytes	No
Anti-IL-6 Abs	No	Scattered diffuse	No	Frequent	No
Anti-TNF-α + Anti-IL-4 Abs	Focal	Moderate	Focal	Frequent	No
Anti-TNF-α + Anti-IFN-γ + Anti-IL-4 Abs	No	Scattered	No	Focal	No
Anti-IL-10 Ab	Minimal	Scattered diffuse	Focal	Focal	No
Anti-TNF-α+ Anti-IL-4 + Anti-IL-10 Abs	No	Moderate	Minimal	Frequent binucleated hepatocytes	No
Anti-IL-4 Abs	Focal	Diffuse	Frequent	Frequent	No
Anti-IFN-γ Ab	No	Scattered	No	Yes	No

H

Cocktail D 1.8X *BALB/c* Group	Proximal Tubular Changes		Distal Tubular Changes		Glomerular changes
	Vacuolation	Degeneration	Desquamation	Foam Cells	Foot Process Effacement
Control IgG	Extensive	Multisegmental	Yes	Yes	Yes
Anti-TNF-α Ab	Rare	Focal	No	Yes	Minimal
Anti-IL-4 Abs	Rare	Focal	Yes	Yes	Segmental
Anti-TNF-α + Anti-IFN-γ + Anti-IL-4 Abs	Frequent	Focal extensive	Yes	Yes	Segmental
Anti-IL-6 Abs	Scattered, extended	Focal	Yes	Yes frequent	Segmental
Anti-IL-10 Ab	Scattered, extended	Focal	No	Yes	Segmental
Anti-TNF-α + Anti-IL-4 Abs	Frequent	Focal moderate	Yes	Yes	Segmental
Anti-TNF-α+ Anti-IL-4 + Anti-IL-10 Abs	Frequent	Focal	Yes	Yes	Segmental
Anti-IFN-γ Ab	Rare	Focal	No	Yes	No

Supplementary Figure 3: Histological sections from studies of *BALB/c* mice (n = 3 mice/group) euthanized 24 hours after Cocktail D dose 1.8x injection and additional antibodies or Control IgG injected one hour after model induction (Figure 3). The numbering code for each group is shown below the image panels. (**A**) Two columns of H & E stained sections of the heart and pericardium. Myocytolysis (red arrows), inflammation (black arrows), hypereosinophilia (green arrows), pericarditis (orange arrow) and pericardial microcalcification (blue arrow) were noted. (**B**) H & E stained sections of the liver. Hepatocellular injury (red arrows), inflammation (black arrows), degenerative changes (green arrows), and regenerative changes (yellow arrows) were noted. (**C**) Toluidine blue stained epon sections of the kidney showing gross tubular morphology. Tubular vacuolation (red arows) and tubular degeneration (black arrows) were noted in proximal tubules. (**D**) Electron microscopy of kidney tubules. Tubular vacuolation (red arows) and tubular degeneration (black arrows) were noted in proximal tubules. (**E**) Electron

microscopy of glomeruli. Areas of podocyte foot process effacement (black arrows) were noted. (**F**) Morphometric analysis and comparison of histological changes in the heart between control IgG and antibody treated Cocktail D 1.8X injected *BALB/c* mice (n = 3 mice / group). (**G**) Morphometric analysis and comparison of histological changes in the liver between control IgG and antibody treated Cocktail D 1.8X injected *BALB/c* mice (n = 3 mice / group). (**H**) Morphometric analysis and comparison of histological changes in the kidney between control IgG and antibody treated Cocktail D 1.8X injected *BALB/c* mice (n = 3 mice / group). Scale bars (a) 20 μm (b) 20 μm (c) 20 μm (d) 0.5 μm (e) 0.5 μm.

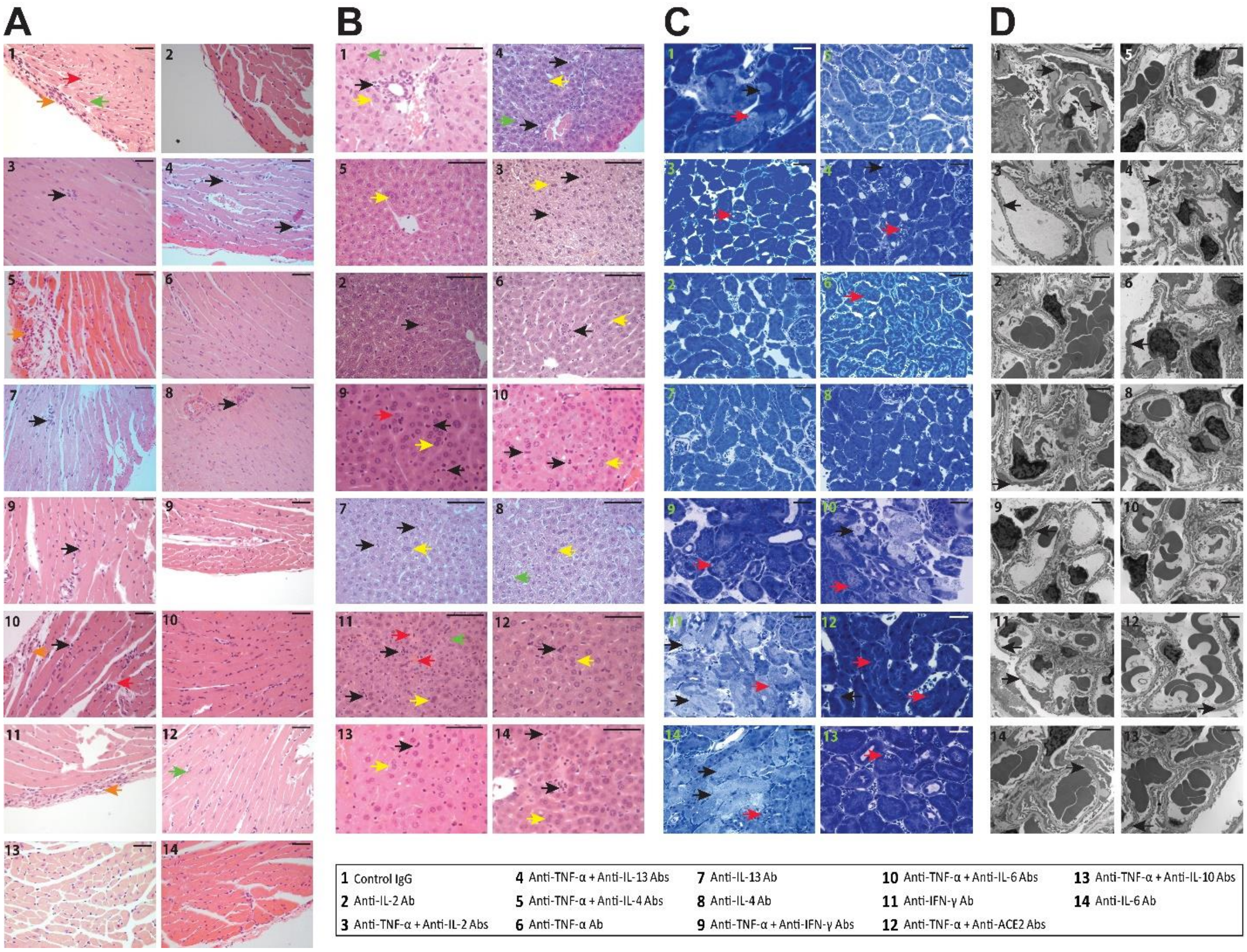

E

Cocktail D 3X *BALB/c* Group	Myocytolysis (Vacuolation)	Hypereosinophilia	Inflammation	Pericarditis
Control IgG	Yes	Focal	Mild	Yes
Anti-IL-2 Ab	No	No	No	No
Anti-TNF-α + Anti-IL-2 Abs	No	No	Minimal	No
Anti-TNF-α + Anti-IL-13 Abs	No	No	Moderate to Severe	No
Anti-TNF-α + Anti-IL-4 Abs	No	No	Minimal	Yes
Anti-TNF-α Ab	No	No	No	No
Anti-IL-13 Ab	No	No	Mild to Moderate	No
Anti-IL-4 Ab	Focal	No	Minimal to Mild	No
Anti-TNF-α + Anti-IFN-γ Abs	Focal	No	Mild	Focal
Anti-TNF-α + Anti-IL-6 Abs	Focal	No	scatttered	Focal
Anti-IFN-γ Ab	Yes	Yes, focal	No	Yes
Anti-TNF-α + Anti-ACE2 Abs	No	Focal	Minimal	No
Anti-TNF-α + Anti-IL-10 Abs	No	No	Minimal	No
Anti-IL-6 Ab	No	No	No	No

F

Cocktail D 3X *BALB/c* Group	Hepatocellular Injury	Inflammation	Degenerative Changes	Regenerative Changes	Peri-central Vein Injury
Control IgG	Yes, marked	Minimal, Prominent Kupfer cells	Frequent	Frequent	No
Anti-TNF-α + Anti-IL-13 Abs	Very Focal	Multifocal	Focal	Frequent	Yes
Anti-TNF-α + Anti-IL-4 Abs	No	Minimal	No	Occasional ballooned hepatocytes	No
Anti-TNF-α + Anti-IL-2 Abs	No	Scattered, Diffuse	No	Frequent	No
Anti-IL-2 Ab	No	Scattered, Minimal	No	Rare	No
Anti-TNF-α Ab	No	Scattered, Minimal	No	Focal	No
Anti-TNF-α + Anti-IFN-γ Abs	Focal	Moderate	Focal	Frequent	No
Anti-TNF-α + Anti-IL-6 Abs	No	Moderate	Minimal	Focal binucleated hepatocytes	No
Anti-IL-13 Ab	No	Moderate, Diffuse	No	Focal binucleated hepatocytes	No
Anti-IL-4 Ab	No	Minimal	Focal	Frequent binucleated hepatocytes	No
Anti-IFN-γ Ab	Marked	Diffuse	Frequent	Frequent	No
Anti-TNF-α + Anti-ACE2 Abs	No	Focal	Focal	Frequent binucleated hepatocytes	No
Anti-TNF-α + Anti-IL-10 Abs	No	Scattered	No	Yes	No
Anti-IL-6 Ab	No	diffuse	No	Frequent	No

G

Cocktail D 3X *BALB/c* Group	Proximal Tubular Changes		Distal Tubular Changes		Glomerular changes
	Vacuolation	Degeneration	Desquamation	Foam Cells	Foot Process effacement
Control IgG	Yes, marked	Yes, multifocal	Yes	Occasional	Extensive
Anti-TNF-α + Anti-IL-4 Abs	No	No	No	No	No
Anti-TNF-α + Anti-IL-2 Abs	Moderate	Yes	No	No	Multisegmental
Anti-TNF-α + Anti-IL-13 Abs	Moderate to Severe	Multifocal	Yes	Yes	Multisegmental
Anti-IL-2 Ab	Minimal	No	No	No	Minimal
Anti-TNF-α Ab	Mild	No	No	No	Segmental
Anti-IL-13 Ab	Mild Apical	No	No	No	Segmental
Anti-IL-4 Ab	Minimal Apical	No	No	No	Minimal
Anti-TNF-α + Anti-IFN-γ Abs	Frequent	Focal	No	Yes	Segmental
Anti-TNF-α + Anti-IL-6 Abs	Rare	Focal moderate	No	Yes	No
Anti-IFN-γ Ab	Extensive	Extensive	Yes	Yes, frequent	Multi-segmental
Anti-TNF-α + Anti-ACE2 Abs	Frequent	Focal	Yes	Yes	Segmental
Anti-IL-6 Ab	Frequent	Focal extensive	Yes	Yes	Segmental
Anti-TNF-α + Anti-IL-10 Abs	Frequent	Focal moderate	Yes	Yes	Segmental

Supplementary Figure 4: Histological sections from studies of *BALB/c* mice (n = 3 mice/group) euthanized 24 hours after Cocktail D dose 3X injection and additional antibodies or Control IgG injected one hour after model induction (Figure 4). The numbering code for each group is shown below the image panels. (**A**) Two columns of H & E stained sections of the heart and pericardium. Myocytolysis (red arrows), inflammation (black arrows), hypereosinophilia (green arrows) and pericarditis (orange arrow) were noted. (**B**) Two columns of H & E stained sections of the liver. Hepatocellular injury (red arrows), inflammation (black arrows), degenerative

changes (green arrows), and regenerative changes (yellow arrows) were noted. (**C**) Two columns of Toluidine blue stained epon sections of the kidney showing gross tubular morphology. Tubular vacuolation (red arows) and tubular degeneration (black arrows) were noted in proximal tubules. (**D**) Two columns of electron microscopy of the kidney showing images of glomeruli. Areas of podocyte foot process effacement (black arrows) were noted. (**E**) Morphometric analysis and comparison of histological changes in the heart between control IgG and antibody treated Cocktail D 3X injected *BALB/c* mice (n = 3 mice / group). (**F**) Morphometric analysis and comparison of histological changes in the liver between control IgG and antibody treated Cocktail D 3X injected *BALB/c* mice (n = 3 mice / group). (**G**) Morphometric analysis and comparison of histological changes in the kidney between control IgG and antibody treated Cocktail D 3X injected *BALB/c* mice (n = 3 mice / group). Scale bars (a) 20 μm (b) 20 μm (c) 20 μm (d) 0.5 μm.

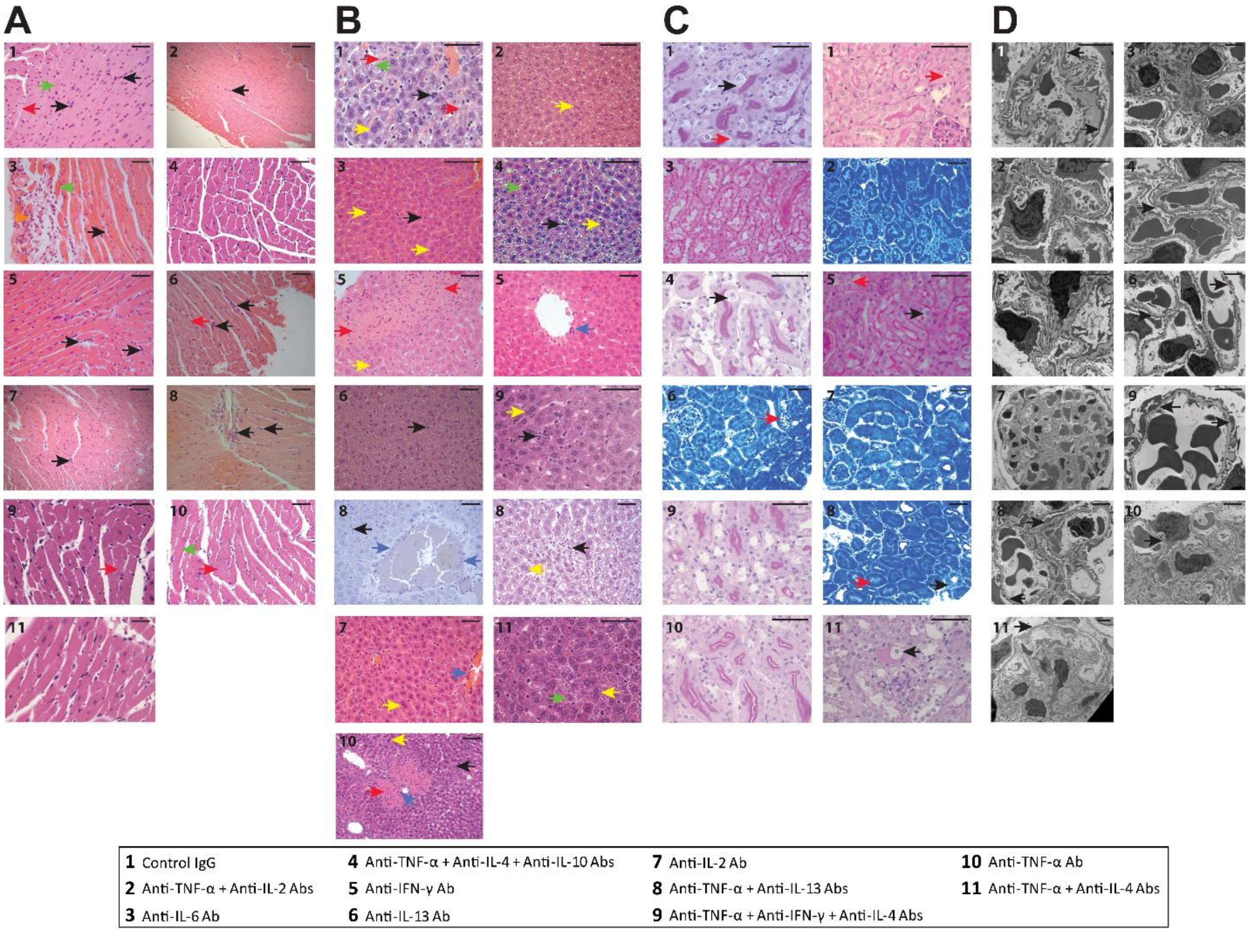

E

Cocktail D 3X *BALB/cJ* Group	Myocytolysis (Vacuolation)	Hypereosinophilia	Inflammation	Pericarditis
Control IgG	Yes	Yes	Moderate	No
Anti-TNF-α + Anti-IL-2 Abs	Very Focal	No	Mild Focal	No
Anti-IL-6 Ab	No	Yes	Moderate	Yes
Anti-TNF-α + Anti-IL-4 + Anti-IL-10 Abs	No	No	Mild scattered	No
Anti-IFN-γ Ab	No	Focal	Multifocal, mild to moderate	Mild focal
Anti-IL-13 Ab	Focal	No	Multifocal	No
Anti-IL-2 Ab	No	No	Minimal	No
Anti-TNF-α + Anti-IL-13 Abs	Very Focal	No	Multifocal	No
Anti-TNF-α + Anti-IFN-γ + Anti-IL-4 Abs	Yes	No	Mild	NA
Anti-TNF-α Ab	Yes	Yes	Minimal, ischemic changes noted	NA
Anti-TNF-α + Anti-IL-4 Abs	Rare	No	Mild scattered	NA

F

Cocktail D 3X *BALB/cJ* Group	Hepatocellular Injury	Inflammation	Degenerative Changes	Regenerative Changes	Peri-central Vein Injury
Control IgG	Extensive	Moderate	Yes	Frequent	Yes
Anti-TNF-α + Anti-IL-2 Abs	No	Minimal	No	Mild	No
Anti-IL-6 Ab	No	Mild	No	Frequent	Yes
Anti-TNF-α + Anti-IL-4 + Anti-IL-10 Abs	No	Mild	Yes	Frequent binucleated, focal ballooned hepatocytes	No
Anti-IFN-γ Ab	Focal Necrosis	Mild	No	Frequent	Mild Segmental
Anti-IL-13 Ab	No	Scattered Moderate	No	Rare	Minimal
Anti-TNF-α + Anti-IFN-γ + Anti-IL-4 Abs	Focal	Focal, Mild	No	Frequent binucleated hepatocytes	No
Anti-TNF-α + Anti-IL-13 Abs	No	Moderate	No	Frequent	Severe
Anti-IL-2 Ab	No	Scattered Mild	No	Mild	Minimal
Anti-TNF-α + Anti-IL-4 Abs	No	Minimal	Yes	Frequent binucleated, focal ballooned hepatocytes	No
Anti-TNF-α Ab	Perivenular ischemic necrosis	Mild to Moderate	Minimal (in areas of necrosis)	Frequent binucleated, focal ballooned hepatocytes	Yes

G

Cocktail D 3X *BALB/cJ* Group	Proximal Tubular Changes		Distal Tubular Changes		Glomerular Changes
	Vacuolation	Degeneration	Desquamation	Foam Cells	Foot Process Effacement
Control IgG	Frequent	Multifocal	Yes	Frequent	Extensive Multisegmental
Anti-IL-6 Ab	Rare	Minimal	No	Yes	Minimal
Anti-TNF-α + Anti-IL-2 Abs	Rare	No	No	No	Minimal
Anti-TNF-α + Anti-IL-4 + Anti-IL-10 Abs	No	Mild	No	No	Segmental
Anti-IFN-γ Ab	Rare	Minimal	No	Yes	Minimal
Anti-IL-13 Ab	Severe	Yes	Yes	Yes	Multisegmental
Anti-IL-2 Ab	Mild	No	No	No	Minimal
Anti-TNF-α + Anti-IFN-γ + Anti-IL-4 Abs	No	No	No	No	Multisegmental
Anti-TNF-α + Anti-IL-13 Abs	Severe apical	Yes	No	No	Multisegmental
Anti-TNF-α Ab	No	Minimal	No	No	Segmental
Anti-TNF-α + Anti-IL-4 Abs	No	Minimal	No	Occasional	Segmental

Supplementary Figure 5: Histological sections from studies of *BALB/cJ* mice (n = 3 mice/group) euthanized 24 hours after Cocktail D dose 3x injection and additional antibodies or Control IgG injected one hour after model induction (Figure 5). The numbering code for each group is shown below the image panels. (**A**) Two columns of H & E stained sections of the heart and pericardium. Myocytolysis (red arrows), inflammation (black arrows), hypereosinophilia (green arrows) and pericarditis (orange arrow) were noted. (**B**) Two columns of H & E stained

sections of the liver. Hepatocellular injury (red arrows), inflammation (black arrows), degenerative changes (green arrows), regenerative changes (yellow arrows), and peri-central vein ischemic injury (blue arrows) were noted. (**C**) Two columns of Toluidine blue stained epon or H & E stained sections of the kidney showing gross tubular morphology. Tubular vacuolation (red arows) and tubular degeneration (black arrows) were noted in proximal tubules. (**D**) Two columns of electron microscopy of the kidney showing images of glomeruli. Areas of podocyte foot process effacement (black arrows) were noted. (**E**) Morphometric analysis and comparison of histological changes in the heart between control IgG and antibody treated Cocktail D 3X injected *BALB/cJ* mice (n = 3 mice / group). NA is Not Assessed. (**F**) Morphometric analysis and comparison of histological changes in the liver between control IgG and antibody treated Cocktail D 3X injected *BALB/cJ* mice (n = 3 mice / group). (**G**) Morphometric analysis and comparison of histological changes in the kidney between control IgG and antibody treated Cocktail D 3X injected *BALB/cJ* mice (n = 3 mice / group). Scale bars (a) 20 μm (b) 20 μm (c) 20 μm (d) 0.5 μm.

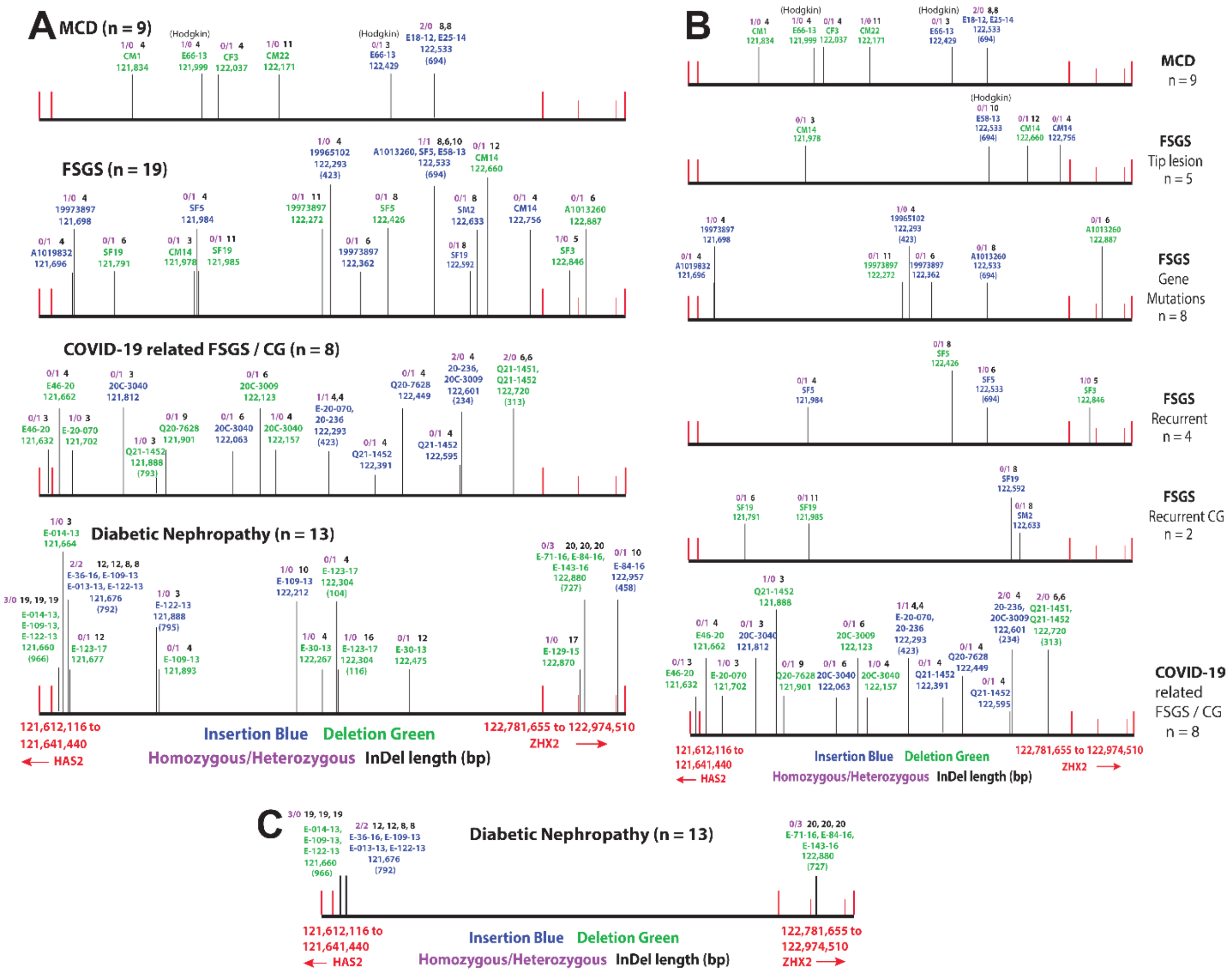

Supplementary Figure 6: (**A**) Single and shared Insertions and Deletions (InDels) in the study population (**B**) InDels in FSGS patients expanded by disease sub-categories. (**C**) Shared InDels in diabetic nephropathy patients (n =13).

A

Disease	Subcategory	Patient ID	Region	InDel	Length	Deletion (Reference)	Insertion (Allele)	Zygosity	Controls positive	Control subjects	1000 Genomes
MCD		CM1	121834023..121834026	Deletion	4	TTTT		Homozygous	0	33	Negative
MCD		CF3	122037539..122037542	Deletion	4	TATT		Heterozygous	0	33	Negative
MCD		CM22	122171109..122171119	Deletion	11	TTTTTTTTTTT	-	Homozygous	0	33	Negative
MCD (Hodgkin)		E66-13	121999676..121999679	Deletion	4	GGAT		Homozygous	0	33	Negative
MCD (Hodgkin)		E66-13	122429110^122429111	Insertion	3	-	AAA	Heterozygous	0	33	Negative
FSGS	Tip lesion	CM14	121978159..121978161	Deletion	3	ATC	-	Heterozygous	0	33	Negative
FSGS	Tip lesion	CM14	122660682..122660693	Deletion	12	GTCACATTCACA	-	Heterozygous	0	33	Negative
FSGS	Tip lesion	CM14	122756989^122756990	Insertion	4	-	TTTG	Heterozygous	0	33	Negative
FSGS	Mutation	A1019832	121696976^121696977	Insertion	4	-	CACA	Heterozygous	0	33	Negative
FSGS	Mutation	19973897	121698198^121698199	Insertion	4	-	AGAG	Homozygous	0	33	Negative
FSGS	Mutation	19973897	122272923..122272933	Deletion	11	TTGTTCAATAT	-	Heterozygous	0	33	Negative
FSGS	Mutation	19973897	122362176^122362177	Insertion	6	-	ACACAA	Heterozygous	0	33	Negative
FSGS	Mutation	A1013260	122887056..122887061	Deletion	6		GTGTGT	Heterozygous	0	33	Negative
FSGS	Recurrent	SF5	121984682^121984683	Insertion	4	-	ATCA	Heterozygous	0	33	Negative
FSGS	Recurrent	SF5	122426275..122426282	Deletion	8		TTTTTTTT	Heterozygous	0	33	Negative
FSGS	Recurrent	SF3	122846550..122846554	Deletion	5	TTTTT	-	Homozygous	0	33	Negative
FSGS	Recurrent CG	SF19	121791036..121791041	Deletion	6	AGAGAG	-	Heterozygous	0	33	Negative
FSGS	Recurrent CG	SF19	121985076..121985086	Deletion	11	CCCAGATCTAG	-	Heterozygous	0	33	Negative
FSGS	Recurrent CG	SF19	122592869^122592870	Insertion	8	-	TCTCTCTC	Heterozygous	0	33	Negative
FSGS	Recurrent CG	SM2	122633967^122633968	Insertion	8	-	TCTCCAAA	Heterozygous	0	33	Negative
COVID-19	CG	46-20	121632761..121632763	Deletion	3	TCA	-	Heterozygous	0	33	Negative
COVID-19	CG	46-20	121662755..121662759	Deletion	4	TTTC	-	Heterozygous	0	33	Negative
COVID-19	CG	E-20-070	121702467..121702469	Deletion	3	TTT	-	Homozygous	0	33	Negative
COVID-19	CG	20C-3040	121812598^121812599	Insertion	3	-	AAA	Heterozygous	0	33	Negative
COVID-19	CG	Q21-1452	121888793..121888802	Deletion	3	TTT	-	Homozygous	0	33	Negative
COVID-19	CG	Q20-7628	121901657..121901665	Deletion	9	TATGCTAGG	-	Heterozygous	0	33	Negative
COVID-19	CG	20C-3040	122063098^122063099	Insertion	6	-	ATATAG	Heterozygous	0	33	Negative
COVID-19	CG	20C-3009	122123611..122123616	Deletion	6	CAACAT	-	Heterozygous	0	33	Negative
COVID-19	CG	20C-3040	122157919..122157922	Deletion	4	AAAA	-	Homozygous	0	33	Negative
COVID-19	CG	Q21-1452	122391579^122391580	Insertion	4	-	ACTG	Heterozygous	0	33	Negative
COVID-19	CG	Q20-7628	122449637^122449638	Insertion	4	-	AGAG	Heterozygous	0	33	Negative
COVID-19	CG	Q21-1452	122595293^122595294	Insertion	4	-	AAAC	Heterozygous	0	33	Negative

B

$ *SHas2* instead of *Has2*

Common Name	Chromosome/Scaffold No.	Annotation release	Assembly	Position on Genome *Has2*		*Slc22a22*			*Zhx2*		Resting Heart Rates (bpm) Taylor, 2005
				Start	End	Start	End	Number of Introns	Start	End	
Mouse	Chromosome 15	109	GRCm39 (GCF_000001635.27)	56529023	56557942	57107163	57341021	12	57558063	57703228	450-750
Rat	Chromosome 7	108	mRatBN7.2 (GCF_015227675.2)	88113326	88139337	88720425	88750111	11	89226358	89374266	250-400
Chicken	Chromosome 2	103	Gallus_gallus-5.0 (GCF_000002315.4)	137467301	137485205	absent	absent	absent	137930168	137997330	250–300
Guinea pig	Unplaced Scaffold	103	Cavpor3.0 (GCF_000151735.1)	625407	650362	absent	absent	absent	12641881	12716872	200-300
Rabbit	Chromosome 3	102	OryCun2.0 (GCF_000003625.3)	137491970	137519857	absent	absent	absent	138688553	138843632	180-350
Rhesus monkey	Chromosome 8	102	Mmul_8.0.1 (GCF_000772875.2)	120797347	120824831	absent	absent	absent	121986575	122179181	160-330
Cat	Chromosome F2	104	Felis_catus_9.0 (GCF_000181335.3)	67278666	67308426	absent	absent	absent	68234407	68397794	120-140
Dog	Chromosome 13	105	CanFam3.1 (GCF_000002285.3)	20310946	20342647	absent	absent	absent	21231985	21393756	70-120
Pig$	Chromosome 4	106	Sscrofa11.1 (GCF_000003025.6)	17583154	17613524	absent	absent	absent	16374900	16553859	70-120
Goat	Chromosome 14	102	ASM170441v1 (GCF_001704415.1)	63935102	63975351	absent	absent	absent	65072984	65253400	70-80
Human	Chromosome 8	109.20211119	GRCh38.p13 (GCF_000001405.39)	121612116	121641440	absent	absent	absent	122781655	122974510	60-80
Cow	Chromosome 14	105	Bos_taurus_UMD_3.1.1 (GCF_000003055.6)	19703611	19733798	absent	absent	absent	18368119	18550996	48-84

Supplementary Figure 7: (**A**) List of single InDels in the study population. (**B**) The *Slc22a22* gene between *Has2* and *Zhx2* in rodents, and its absence in larger animals and humans, arranged by size and heart rate. Heart rate data derived from Taylor, R. Dukes' Physiology of Domestic Animals. 12th edition, William O. Reece, Editor. Cornell University Press, Ithaca, 2004.

A

Disease	Patient ID	Region	InDel	Length	Deletion (Reference)	Insertion (Allele)	Zygosity	Sample Count (case)	Controls positive	Control subjects	1000 Genomes
Diabetic Nephropathy	E-014-13, E-109-13, E-122-13	121660966..121660984	Deletion	19	TTCATCAGTATTTTCTTTC	-	Homozygous	3	0	33	Negative
Diabetic Nephropathy	E-36-16, E-109-13	121676792^121676793	Insertion	12	-	TTTATTTATTTA	Homozygous	2	0	33	Negative
Diabetic Nephropathy	E-013-13, E-122-13	121676792^121676793	Insertion	8	-	TTTATTTA	Heterozygous	2	0	33	Negative
Diabetic Nephropathy	E-71-16, E-84-16, E-143-16	122880727..122880746	Deletion	20	ATCATGCCACTACACTCCAG	-	Heterozygous	3	0	33	Negative

B

Disease	Patient ID	Region	InDel	Length	Deletion (Reference)	Insertion (Allele)	Zygosity	Sample Count (case)	Controls positive	Control subjects	1000 Genomes
Diabetic Nephropathy	E-014-13	121664218..121664220	Deletion	3	GGA	-	Homozygous	1	0	33	Negative
Diabetic Nephropathy	E-123-17	121677501..121677512	Deletion	12	TGTGTGTGTGTG	-	Heterozygous	1	0	33	Negative
Diabetic Nephropathy	E-122-13	121888795^121888796	Insertion	3	-	TTT	Homozygous	1	0	33	Negative
Diabetic Nephropathy	E-109-13	121893131..121893134	Deletion	4	GAAA	-	Heterozygous	1	0	33	Negative
Diabetic Nephropathy	E-109-13	122212772^122212773	Insertion	10	-	ATGCAAATTA	Homozygous	1	0	33	Negative
Diabetic Nephropathy	E-30-13	122267042..122267045	Deletion	4	TCAT	-	Homozygous	1	0	33	Negative
Diabetic Nephropathy	E-123-17	122304014..122304017	Deletion	4	ACTT	-	Heterozygous	1	0	33	Negative
Diabetic Nephropathy	E-84-16	122304116..122304131	Deletion	16	GGATGGATGGATGGAT	-	Homozygous	1	0	33	Negative
Diabetic Nephropathy	E-30-13	122475107..122475118	Deletion	12	GATAGATAGGTA	-	Heterozygous	1	0	33	Negative
Diabetic Nephropathy	E-129-15	122870639..122870655	Deletion	17	AAAAAAAAAAAAAAAAA	-	Homozygous	1	0	33	Negative
Diabetic Nephropathy	E-84-16	122957458^122957459	Insertion	10	-	TTGTTTTGTT	Heterozygous	1	0	33	Negative

C

Disease	Patient ID	Region	InDel	Length	Deletion (Reference)	Insertion (Allele)	Zygosity	Sample count (case)	Controls positive	Control subjects	1000 Genomes
Diabetic Nephropathy	E-122-13	121888795^121888796	Insertion	3	-	TTT	Homozygous	1	0	33	Negative
COVID-19 CG	Q21-1452	121888793..121888795	Deletion	3	TTT	-	Homozygous	1	0	33	Negative

D

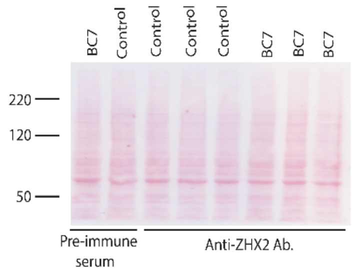

Supplementary Figure 8: (**A**) List of shared InDels in diabetic patients. (**B**) List of single InDels in diabetic patients. (**C**) Common InDel site between a diabetic and COVID-19 CG patient. (**D**) Ponceau Red stained membrane from blot shown in Fig. 6E.

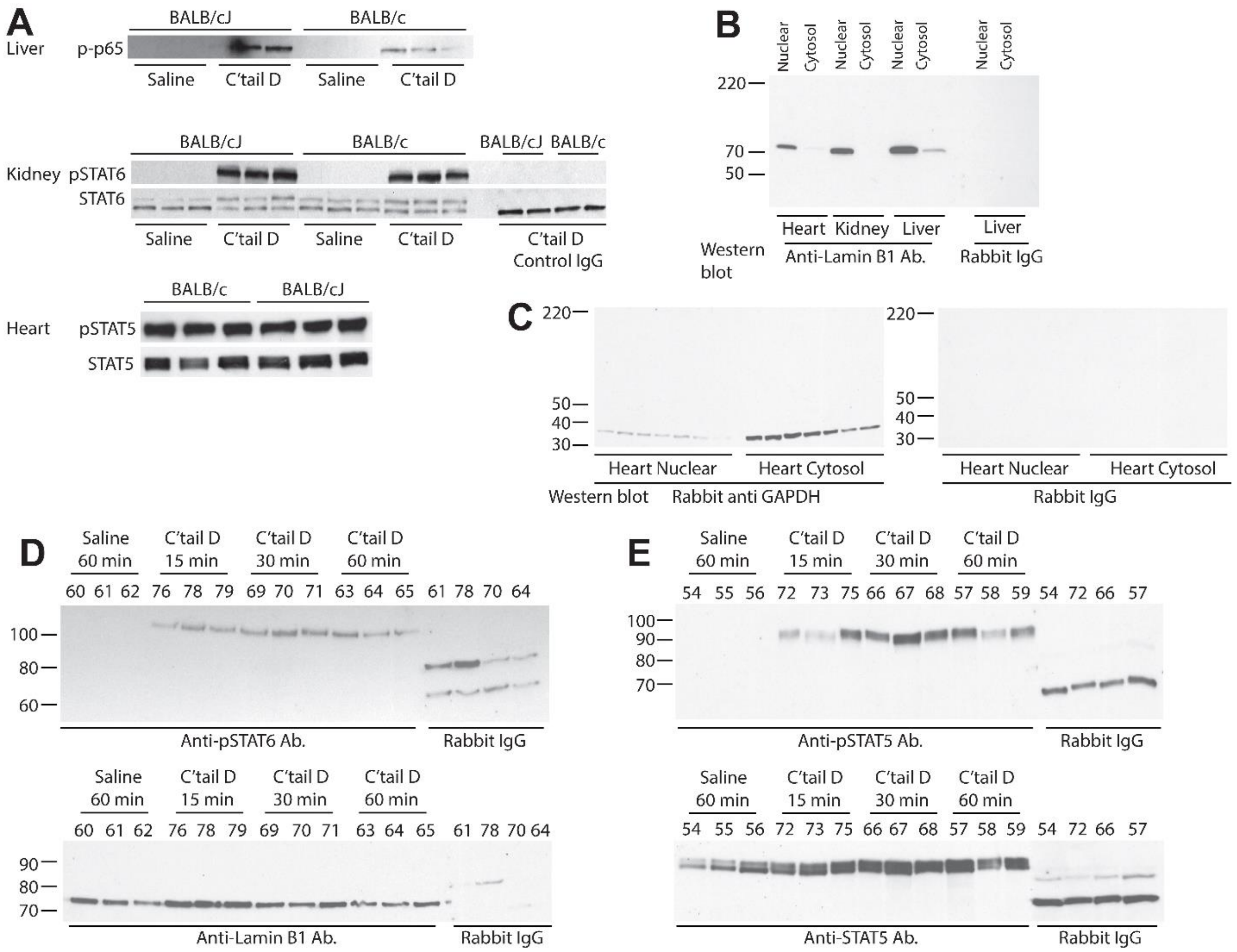

Supplementary Figure 9: (**A**) Qualitative studies with examples of NFκB / p-p65 (liver, 30 minutes), pSTAT6 (kidney 60 minutes) and pSTAT5 (heart, 15 minutes) activation by Western blot of protein extracts of mice (n = 3 per group) injected with Cocktail D 3X or control saline. (**B**) Examples of nuclear extract purity studies by Western blot of nuclear and cytosol fractions with equal protein loading from heart, kidney and liver using anti-Lamin B1 antibody. Traces of Lamin B1 in the cytosol may be related to protein synthesis prior to transport to the nucleus. (**C**) Examples of assessment of GAPDH protein in heart cytosolic and nuclear fractions with equal protein loading from multiple mice. As expected, GAPDH is expressed in both fractions, with greater expression in cytosolic extracts. (**D**) Quantitative studies showing example of Western blots of *BALB/c* mouse heart nuclear protein extracts (20 μg protein per lane) in a Cocktail D (C'tail D) or control saline injection study assessed for pSTAT6 and Lamin B1 on separate blots

developed on the same film. Abbreviated mouse numbers are shown for each lane. (**E**) Quantitative studies showing example of Western blots of *BALB/cJ* mouse heart cytosolic protein extracts (20 μg protein per lane) in a Cocktail D (C'tail D) or control saline injection study assessed for pSTAT5 and STAT5 on separate blots developed on the same film. Abbreviated mouse numbers are shown for each lane.

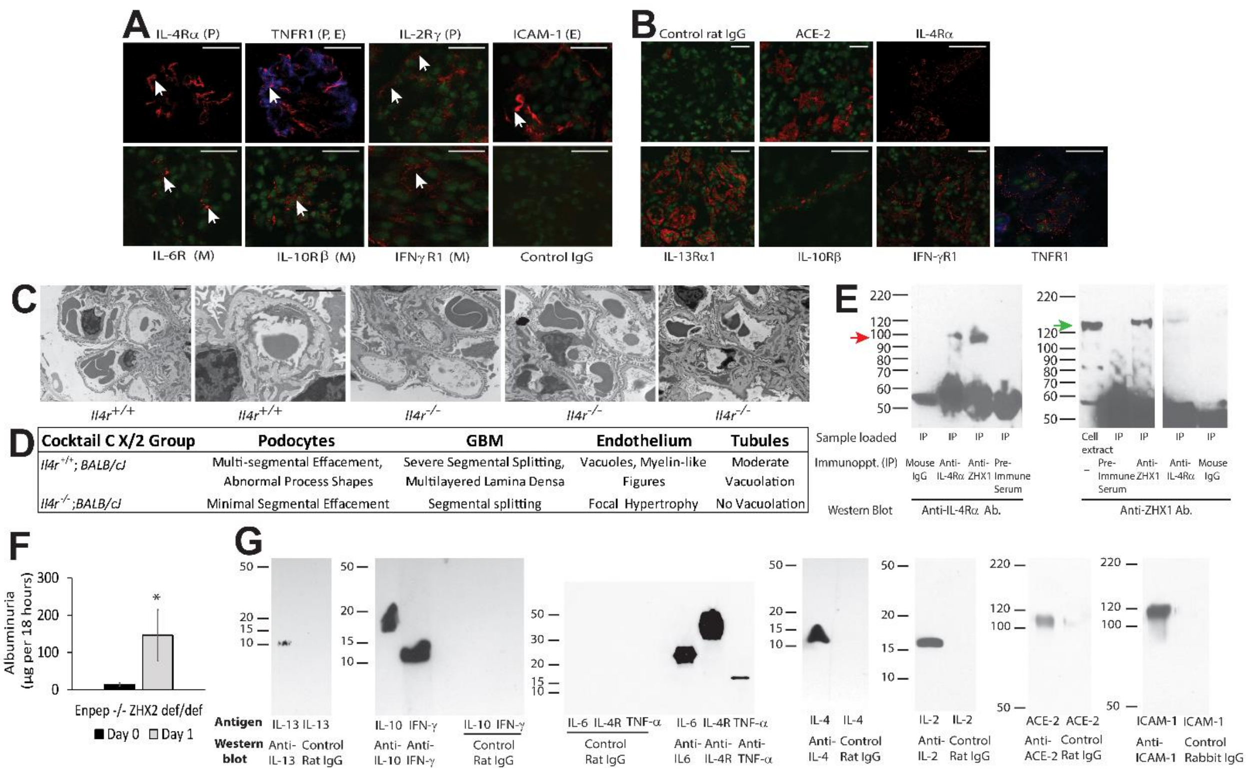

Supplementary Figure 10: (**A**) Confocal expression of cytokine receptors in *BALB/c* mouse glomeruli. White arrows indicate receptor expression in podocytes (P), endothelial (E) and mesangial (M) cells. Since TNFR1 is expressed in podocytes and endothelial cells, only partial co-localization with nephrin (blue), a podocyte protein, is noted. Green color is nuclear stain. (**B**) Confocal expression (red) of ACE-2 and cytokine receptors in *BALB/c* mouse kidney tubules. Most images show proximal tubules, except IL-10Rβ image is collecting duct. (**C**) Electron microscopy images of glomeruli from mice in Fig. 8B. (**D**) Morphometric analysis of kidneys of mice from Fig. 8B (n = 3 mice/group). (**E**) Reducing SDS PAGE and Western blots from co-immunoprecipitation (co-IP) studies with protein extracted from the CRISPR B human podocyte cell line. Anti-ZHX1 antibody (Ab.) could co-IP IL-4Rα (red arrow), and anti-IL-4Rα could co-IP ZHX1 (green arrow) selectively from this cell line. (**F**) At Common Cold cocktail dose X/2, albuminuria was higher in dual *Zhx2*$^{def/def}$, *Enpep*$^{-/-}$ mice than *BALB/cJ* shown in Fig. 1D. Data represent mean ± SEM. (**G**) Characterization of antibodies used for depletion studies using

recombinant proteins that make up the cytokine cocktails. Scale bars (b) 20 μm (c) 20 μm (d) 0.5 μm.

A

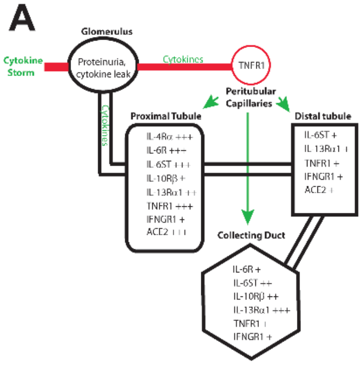

B

Receptor	Cardiac muscle cell type			
	Cardiomyocyte	Fibroblast	Endothelium	Smooth Muscle
IL-4Rα			3+	2+
IL-6R	1+	2+	1+	3+
IL-6ST		1+	3+	1+
IL-10Rβ	1+	1+	3+	1+
IL-13Rα1	trace	1+	3+	2+
TNFR1			3+	1+
IFNGR1		1+	3+	1+
ACE-2		2+		3+

C

Receptor	Liver cell type					
	Hepatocyte	Kupfer cell	Ito cell	Endothelium	Cholangiocyte	Erythroid cell
IL-4Rα	1+	1+	2+	3+	1+	
IL-2Rα		2+			2+	
IL-6R	3+	2+			2+	
IL-6ST	trace		3+	2+	1+	
IL-10Rβ		3+	2+		1+	1+
IL-13Rα1	1+	2+	2+	3+	1+	
TNFR1	1+	1+	2+	3+	1+	
IFNGR1		3+	1+	1+	trace	
ACE-2					3+	

Supplementary Figure 11: Schematic representation of data assembled from the human protein atlas project[42] showing approximate distribution and semi-quantitative expression of cytokine receptors and ACE2 in (**A**) kidney tubular segments (**B**) heart muscle and (**C**) liver.

SUPPLEMENTARY TABLES

Supplementary Table 1: Source of human genomic DNA for study population. DNA samples from Duke University were used for prior (unrelated) studies (52, 53).

Source code	Age or age of onset	Sex	Race	DNA source	Other known features	IRB approval
<u>**MCD patients**</u>						
E-109-12	30	F	Hispanic - Mexico	Native kidney biopsy		Instituto Nacional De Cardiologia, Mexico City
E-18-12	18	F	Hispanic - Mexico	Native kidney biopsy		Instituto Nacional De Cardiologia, Mexico City
E-25-14	26	M	Hispanic - Mexico	Native kidney biopsy		Instituto Nacional De Cardiologia, Mexico City
CM22	29	M	Hispanic - Brazil	EBV immortalized monocytes		University of Alabama at Birmingham
CM17	19	M	African American	EBV immortalized monocytes		University of Alabama at Birmingham
CF12	32	F	Caucasian	EBV immortalized monocytes		University of Alabama at Birmingham
CF3	51	F	Caucasian	EBV immortalized monocytes		University of Alabama at Birmingham
CM1	52	M	Caucasian	EBV immortalized monocytes		University of Alabama at Birmingham
<u>**FSGS patients**</u>						
CM14	46	M	Caucasian	EBV immortalized monocytes	Tip lesion	University of Alabama at Birmingham
E-173-13	53	M	Hispanic - Mexico	Native kidney biopsy	Tip lesion, sclerotic	Instituto Nacional De Cardiologia, Mexico City
19965079-19	14-24		Caucasian	Whole blood or saliva	INF2 exon mutation, published ID 6505	Duke University
19971272-16	19-24		Caucasian-New Zealand	Whole blood or saliva	INF2 exon mutation, published ID 6518	Duke University
19973897-17	4		Caucasian	Whole blood or saliva	NPHS2 exon mutation, published ID 6517	Duke University
A1013260-13	1.5		Caucasian	Whole blood or saliva	WT1 exon mutation, published ID 6659	Duke University
A1019832-13	20		Hispanic-Uruguay	Whole blood or saliva	INF2 exon mutation, published ID 6635	Duke University
A1013255-09	4		Caucasian	Whole blood or saliva	NPHS2 exon mutation,published ID 6647	Duke University
A1034780-10	13		Caucasian	Whole blood or saliva	NPHS2 exon mutation, published ID 34443	Duke University
19965102-19	14-24		Caucasian	Whole blood or saliva	INF2 exon mutation, published ID 6502	Duke University
SF5	46	F	African American	EBV immortalized monocytes	Recurrent FSGS	University of Alabama at Birmingham
SF3	53	F	African American	EBV immortalized monocytes	Recurrent FSGS	University of Alabama at Birmingham
SF18	44	F	African American	EBV immortalized monocytes	Recurrent FSGS	University of Alabama at Birmingham
SM7	44	M	African American	EBV immortalized monocytes	Recurrent FSGS	University of Alabama at Birmingham
SF19	40	F	African American	EBV immortalized monocytes	Recurrent non-HIV collapsing glomerulopathy	University of Alabama at Birmingham
SM2	55	M	Caucasian	EBV immortalized monocytes	Recurrent non-HIV collapsing glomerulopathy	University of Alabama at Birmingham
<u>**Hodgkin Disease**</u>						
E-66-13	26	M	Hispanic - Mexico	Native kidney biopsy	MCD. Hodgkin disease	Instituto Nacional De Cardiologia, Mexico City
E-299-11	30	M	Hispanic - Mexico	Native kidney biopsy	FSGS Tip lesion, cellular. Hodgkin disease	Instituto Nacional De Cardiologia, Mexico City
E-58-13	16	F	Hispanic - Mexico	Native kidney biopsy	FSGS Tip lesion, cellular. Hodgkin disease	Instituto Nacional De Cardiologia, Mexico City
E-114-12	29	M	Hispanic - Mexico	Native kidney biopsy	FSGS Tip lesion, sclerotic. B cell lymphoma / Hodgkin	Instituto Nacional De Cardiologia, Mexico City
<u>**COVID-19 patients**</u>						
20-236	36	M	Hispanic - Peru	Native kidney biopsy	FSGS / Non-HIV collapsing glomerulopathy	Hospital Nacional Alberto Sabogal Essalud, Lima
E46-20	52	M	Hispanic - Mexico	Native kidney biopsy	FSGS / Non-HIV collapsing glomerulopathy	Instituto Nacional De Cardiologia, Mexico City
20C-3040	32	F	Hispanic - Peru	Native kidney biopsy	FSGS / Non-HIV collapsing glomerulopathy	Hospital Nacional Alberto Sabogal Essalud, Lima
20C-3009	43	M	Hispanic - Peru	Native kidney biopsy	FSGS / Non-HIV collapsing glomerulopathy	Hospital Nacional Alberto Sabogal Essalud, Lima
Q21-1452	48	M	Hispanic - Peru	Native kidney biopsy	FSGS / Non-HIV collapsing glomerulopathy	Hospital Nacional Alberto Sabogal Essalud, Lima
Q21-1451	43	M	Hispanic - Peru	Native kidney biopsy	FSGS / Non-HIV collapsing glomerulopathy	Hospital Nacional Alberto Sabogal Essalud, Lima
Q20-7628	24	F	Hispanic - Peru	Native kidney biopsy	FSGS / Non-HIV collapsing glomerulopathy	Hospital Nacional Alberto Sabogal Essalud, Lima
E-20-070			Hispanic - Mexico	Native kidney biopsy	FSGS / Non-HIV collapsing glomerulopathy	Instituto Nacional De Cardiologia, Mexico City
<u>**Controls**</u>						
E07-14	30	M	Hispanic - Mexico	Pre-implantation kidney biopsy	Living donor kidney	Instituto Nacional De Cardiologia, Mexico City
E11-14	45	F	Hispanic - Mexico	Pre-implantation kidney biopsy	Living donor kidney	Instituto Nacional De Cardiologia, Mexico City
E16-14	31	F	Hispanic - Mexico	Pre-implantation kidney biopsy	Living donor kidney	Instituto Nacional De Cardiologia, Mexico City
E18-14	?	M	Hispanic - Mexico	Pre-implantation kidney biopsy	Living donor kidney	Instituto Nacional De Cardiologia, Mexico City
E59-14	30	F	Hispanic - Mexico	Pre-implantation kidney biopsy	Living donor kidney	Instituto Nacional De Cardiologia, Mexico City
E116-14	23	M	Hispanic - Mexico	Pre-implantation kidney biopsy	Living donor kidney	Instituto Nacional De Cardiologia, Mexico City
E121-14	30	M	Hispanic - Mexico	Pre-implantation kidney biopsy	Living donor kidney	Instituto Nacional De Cardiologia, Mexico City
E146-14	44	M	Hispanic - Mexico	Pre-implantation kidney biopsy	Living donor kidney	Instituto Nacional De Cardiologia, Mexico City
E181-14	22	M	Hispanic - Mexico	Pre-implantation kidney biopsy	Living donor kidney	Instituto Nacional De Cardiologia, Mexico City
E189-14	23	M	Hispanic - Mexico	Pre-implantation kidney biopsy	Living donor kidney	Instituto Nacional De Cardiologia, Mexico City
E191-14	30	M	Hispanic - Mexico	Pre-implantation kidney biopsy	Living donor kidney	Instituto Nacional De Cardiologia, Mexico City
E193-14	40	M	Hispanic - Mexico	Pre-implantation kidney biopsy	Living donor kidney	Instituto Nacional De Cardiologia, Mexico City
E200-14	21	M	Hispanic - Mexico	Pre-implantation kidney biopsy	Living donor kidney	Instituto Nacional De Cardiologia, Mexico City
61-18			Hispanic - Mexico	Pre-implantation kidney biopsy	Living donor kidney	Instituto Nacional De Cardiologia, Mexico City
75-19			Hispanic - Mexico	Pre-implantation kidney biopsy	Living donor kidney	Instituto Nacional De Cardiologia, Mexico City
90-19			Hispanic - Mexico	Pre-implantation kidney biopsy	Living donor kidney	Instituto Nacional De Cardiologia, Mexico City
E-41-21	36	F	Hispanic - Mexico	Pre-implantation kidney biopsy	Living donor kidney	Instituto Nacional De Cardiologia, Mexico City
E-42-21	27	M	Hispanic - Mexico	Pre-implantation kidney biopsy	Living donor kidney	Instituto Nacional De Cardiologia, Mexico City
Q21-1545-1-17	47	F	Hispanic - Peru	Pre-implantation kidney biopsy	Living donor kidney	Hospital Nacional Alberto Sabogal Essalud, Lima
NA19750			Mexican Ancestry in Los Angeles	Archived DNA samples	HAPMAP project	HAPMAP / NHGRI
NA19746			Mexican Ancestry in Los Angeles	Archived DNA samples	HAPMAP project	HAPMAP / NHGRI
NA19732			Mexican Ancestry in Los Angeles	Archived DNA samples	HAPMAP project	HAPMAP / NHGRI
NA19728			Mexican Ancestry in Los Angeles	Archived DNA samples	HAPMAP project	HAPMAP / NHGRI
NA19984			African Ancestry in Southwest USA	Archived DNA samples	HAPMAP project	HAPMAP / NHGRI
NA19713			African Ancestry in Southwest USA	Archived DNA samples	HAPMAP project	HAPMAP / NHGRI
NA19922			African Ancestry in Southwest USA	Archived DNA samples	HAPMAP project	HAPMAP / NHGRI
NA19921			African Ancestry in Southwest USA	Archived DNA samples	HAPMAP project	HAPMAP / NHGRI
HG00104			British from England and Scotland UK	Archived DNA samples	1000 genomes project	1000 genomes / NHGRI
HG00115			British from England and Scotland UK	Archived DNA samples	1000 genomes project	1000 genomes / NHGRI
HG00146			British from England and Scotland UK	Archived DNA samples	1000 genomes project	1000 genomes / NHGRI
NA20586			Toscani in Italia	Archived DNA samples	HAPMAP project	HAPMAP / NHGRI
NA20774			Toscani in Italia	Archived DNA samples	HAPMAP project	HAPMAP / NHGRI
NA20805			Toscani in Italia	Archived DNA samples	HAPMAP project	HAPMAP / NHGRI

Supplementary Table 2: Patient characteristics of archived diabetic nephropathy kidney biopsies used to extract genomic DNA.

Source code	Age (years) at biopsy	Sex	Race	DNA source	Proteinuria	IRB approval
E-122-17	64	F	Hispanic - Mexico	Native kidney biopsy	Sub - neprotic	Instituto Nacional De Cardiologia, Mexico City
E-36-16	44	M	Hispanic - Mexico	Native kidney biopsy	Nephrotic (20 grams)	Instituto Nacional De Cardiologia, Mexico City
E-129-15	59	M	Hispanic - Mexico	Native kidney biopsy	Sub-nephrotic (1 gram)	Instituto Nacional De Cardiologia, Mexico City
E-123-17	61	M	Hispanic - Mexico	Native kidney biopsy	Nephrotic (5 grams)	Instituto Nacional De Cardiologia, Mexico City
E-71-16	59	M	Hispanic - Mexico	Native kidney biopsy	500 mg/dl	Instituto Nacional De Cardiologia, Mexico City
E-84-16	29	F	Hispanic - Mexico	Native kidney biopsy	3 grams	Instituto Nacional De Cardiologia, Mexico City
E-143-16	64	M	Hispanic - Mexico	Native kidney biopsy	Nephrotic range	Instituto Nacional De Cardiologia, Mexico City
E-133-15	55	F	Hispanic - Mexico	Native kidney biopsy	8 grams	Instituto Nacional De Cardiologia, Mexico City
E-109-13	50	M	Hispanic - Mexico	Native kidney biopsy	Nephrotic range	Instituto Nacional De Cardiologia, Mexico City
E-122-13	63	F	Hispanic - Mexico	Native kidney biopsy	6 grams	Instituto Nacional De Cardiologia, Mexico City
E-014-13	57	F	Hispanic - Mexico	Native kidney biopsy	1 gram	Instituto Nacional De Cardiologia, Mexico City
E-013-13	51	M	Hispanic - Mexico	Native kidney biopsy	1 gram	Instituto Nacional De Cardiologia, Mexico City
E-30-13	62	M	Hispanic - Mexico	Native kidney biopsy	Nephrotic (10 grams)	Instituto Nacional De Cardiologia, Mexico City

Supplementary Table 3: List of cytokine cocktail components, and antibodies injected.

Cytokine	Mouse		Rat	
	Company	Catalog #	Company	Catalog #
IL-2	R&D Systems	402-ML-100/CF	Sigma-Aldrich	SRP3242-20UG
IL-4Rα	R&D Systems	530-MR-100	Sino Biological	80198-R08H
IL-4	R&D Systems	404-ML-050	R&D Systems	504-RL-025/CF
IL-13	R&D Systems	413-ML-050	R&D Systems	1945-RL-025/CF
IL-6	Sigma	SRP3330-10UG	R&D Systems	506-RL-010/CF
IL-10	Sigma	I3019-5UG	R&D Systems	522-RLB-025/CF
IL-10	Thermo-Fisher	RMIL105		
Inteferon-γ	Millipore	IF005	R&D Systems	585-IF-100
TNF-α	Sigma	T7539-50UG	Sigma-Aldrich	T5944-50UG
ACE-2	R&D Systems	3437-ZN-010	R&D Systems	4516-ZN-010
ICAM-1	R&D Systems	796-IC-050	R&D Systems	583-IC-050

Cytokine/Receptor	Human		Injected antibodies	
	Company	Catalog #	Company	Catalog #
IL-2	R&D Systems	202-IL-010	R&D Systems	MAB702
IL-4Rα	Sino Biological	10402-H08H	R&D Systems	MA**B530**
IL-4	R&D Systems	6507-IL/CF	R&D Systems	MAB404
IL-13			R&D Systems	MAB413
IL-6	R&D Systems	206-IL-010	R&D Systems	MAB406
IL-10	Sino Biological	10947-HNAE	R&D Systems	MAB417
Inteferon-γ	Sino Biological	11725-HNAS	R&D Systems	MAB485
TNF-α	R&D Systems	210-TA-005	R&D Systems	MAB4101
ACE-2	R&D Systems	933-ZN	R&D Systems	MAB3437
ICAM-1	R&D Systems	ADP4-050		
TNFRI			R&D Systems	MAB430
IL-10Rβ			R&D Systems	MAB53681
Rat gamma globulin			Jackson ImmunoResearch	012-000-002
Syrian hamster gamma globulin			Jackson ImmunoResearch	007-000-002

Supplementary Table 4: List of primers and probes.

Name	Application	Primer/ Probe Sequence
	**Generation of Crispr-cas9 mutants**	
	**CRISPR B**	
**G0016**	SgRNA generation	5'-CAC CGA CTG GTA AAC CAC TTA GGG C-3'
**G0017**	SgRNA generation	5'-AAA CGC CCT AAG TGG TTT ACC AGT C-3'
**K1145**	SgRNA plasmid sequencing	5'-GCA TAT ACG ATA CAA GGC TGT TAG AGA G-3'
**K1195**	Donor plasmid	5'-CGG GCC GGA TCC CTA GAT GTA GCA TTA CCA GGG TGG-3'
**K1196**	Donor plasmid	5'-GGC CGA AGC TTG CAG AGA AGA TCA CGA TAG ATT AGA AGA TG-3'
**K1207**	Sequencing of donor plasmid	5'-GGT TTC CTT GTT ATA TCA CCA G-3'
**K1215**	Quickchange Mutagenesis	5'-GCT CTA GGA TGA CTG GTA AAC CAC TTA GGG CAG TCG TCC CCA GAC CTG GTC TGT GGC CTG TTA G-3'
**K1216**	Quickchange Mutagenesis	5'-CTA ACA GGC CAC AGA CCA GGT CTG GGG ACG ACT GCC CTA AGT GGT TTA CCA GTC ATC CTA GAG C-3'
**K1219**	Plasmid linear amplification	5'-GAT TAT CTT TCT AGG GTT AAC GAA CTT CAA GTA ATC AAG AGC AGC-3'
**K1220**	Plasmid linear amplification	5'-CGC AGA CTA TCT TTC TAG GGT TAA CTT TGT AGA ATG CTT CTC G-3'
**K1217**	Puromycin cassette	5'-CGA GAA GCA TTC TAC AAA GTT AAC CCT AGA AAG ATA GTC TGC G-3'
**K1218**	Puromycin cassette	5'-GCT GCT CTT GAT TAC TTG AAG TTC GTT AAC CCT AGA AAG ATA ATC-3'
**K1189**	Genome editing	5'-ACA CTG ACG ACA TGG TTC TAC AGT CTC TGA AAC ATA GAA GGC AC-3'
**K1188**	Genome editing	5'-TAC GGT AGC AGA GAC TTG GTC TGA GAA TCT AAT ACC GCT GAT CTG-3'
	**CRISPR A**	
**G0003**	SgRNA generation	5'-CAC CGA CCC ATC CAT ACA CTT ACC C-3'
**G0004**	SgRNA generation	5'-AAA CGG GTA AGT GTA TGG ATG GGT C-3'
**K1145**	SgRNA plasmid sequencing	5'-GCA TAT ACG ATA CAA GGC TGT TAG AGA G-3'
**K1140**	Donor plasmid	5'-GGC GGC ACT AGT CTA GCT GGC TTG ACT TTA CAA GAC GAT TCC ATC C-3'
**K1141**	Donor plasmid	5'-GGG CGG ATC CCT GCA CTC AGT ATT CTG CAA GTC CTG TAG C-3'
**K1151**	Sequencing of donor plasmid	5'-CGA TCT CCT GAC CTC AAG-3'
**K1149**	Quickchange Mutagenesis	5'-GTG CCT GGC CTG TTA TGA TCT TCT TAC TCA TTT GAT AGC ACC AGT GTC CTG AGA AAA ATA ACA TAT ACT CCA TTA CCC ATC CAT ACA CTT ACC CAG GCA CTC ATT CAC CAT ATT AAC TAG ATA GAC ACA TGA TGT TGC TGC TCC TGT TGA TGA TAA CAA TGT TGA GG-3'
**K1150**	Quickchange Mutagenesis	5'-CCT CAA CAT TGT TAT CAT CAA CAG GAG CAG CAA CAT CAT GTG TCT ATC TAG TTA ATA TGG TGA ATG AGT GAC TGG GTA AGT GTA TGG ATG GGT AAT GGA GTA TAT GTT ATT TTT CTC AGG ACA CTG GTG CTA TCA AAT GAG TAA GAA GAT CAT AAC AGG CCA GGA C-3'
**K1163**	Plasmid linear amplification	5'-CGT CAC AAT ATG ATT ATC TTT CTA GGG TTA ACT AGA TAG ACA CAT GAT GTT GCT GCT CC-3'
**K1164**	Plasmid linear amplification	5'-CGT CAA TTT TAC GCA GAC TAT CTT TCT AGG GTT AAT ATG GTG AAT GAG TGA CTG GG-3'
**K1153**	Puromycin cassette	5'-CCC AGT CAC TCA TTC ACC ATA TTA ACC CTA GAA AGA TAG TCT GCG TAA AAT TGA CG-3'
**K1154**	Puromycin cassette	5'-GGA GCA GCA ACA TCA TGT GTC TAT CTA GTT AAC CCT AGA AAG ATA ATC ATA TTG TGA CG-3'
**K1138**	Genome editing	5'-ACA CTG ACG ACA TGG TTC TAC AGT TAT GAT CTT CTT ACT CAT TTG ATA GCA CCA GTG TCC-3'
**K1139**	Genome editing	5'-TAC GGT AGC AGA GAC TTG GTC TGA AAG GAG CAG TGT TGA TCT AGA GAG AGC C-3'
	**Real time PCR**	
**H826**	Human ZHX2 forward primer	CGGAACTGGCTGAATCAGACT
**H827**	Human ZHX2 reverse primer	CAGCACAGCAGTTCTAACAGACTT
**P246**	FAM-MGB Probe	TGCAGAGGCTGGCCA

Online Methods

Mass Spectrometry assay for plasma creatinine

Serum creatinine was measured by LC/MS/MS using an Agilent 1290 Infinity II LC system in combination with a 2x50mm, 2 µm Tosoh Bioscience TSK-GEL amide-80 LC column, interfaced to an Agilent 6495 Triple Quadrupole. The oven temperature was fixed at 40˚C. The mobile phase consisted of 10mM ammonium acetate in LCMS-grade water (35%) and LCMS-grade acetonitrile (ACN; 65%). Synthetic creatinine (ranging from 20 µg/ml to 0.16 ug/ml; Sigma-Aldrich, Inc, St. Louis MO) and isotope-labeled creatinine (D_3-creatinine, 10 µg/ml; Sigma-Aldrich) were used as standard and internal standard, respectively. Then, 10 ul of sample or standard was combined with 5 µl internal standard and 235 ul 100% ACN, vortexed and centrifuged at 4˚C for 15 min at 15000 rpm. The supernatant was transferred to a new tube with 200 µl 10 mM ammonium acetate and 65% acetonitrile in LCMS-grade water, vortexed, centrifuged at 4˚C for 15 min at 15000 rpm and subsequently measured. All samples were measured in duplicate.

Genome editing in cultured human podocytes using CRISPR/Cas9

A single cell derived clone of cells was generated from an established early passage immortalized human podocyte cell line (60) and used for genome editing studies. The oligonucleotides and primers used are listed in **Supplementary Table 4**.

CRISPR B

Generation of the sgRNA plasmid: In order to introduce a 10 bp insertion (CACACACACA), sgRNA recognizing a specific site 45 bp downstream of the insertion site (Chr8-122,533,694 - 122,533,695) was designed using the Benchling website (https://benchling.com). Oligos G0016 and G0017 were phosphorylated and annealed using T4

Polynucleotide Kinase (New England Biolabs, Ipswich MA), digested with *Bbs*I and ligated into pX330-U6-Chimeric_BB-CBh-hSpCas9 plasmid (a gift from Feng Zhang, Addgene plasmid # 42230) using T7 DNA ligase (New England Biolabs). The ligation product was treated with PlasmidSafe exonuclease (Epicentre /Illumina San Diego CA) to prevent unwanted recombination products and then transformed into One Shot TOP10 cells (Invitrogen / Thermo Fisher Scientific, Waltham MA). Ten colonies were picked up and plasmids were isolated using QIAprep Spin Miniprep Kit (QIAgen, Hilden Germany). Plasmid DNA was sequenced using primer K1145.

Generation of the donor plasmid: The human genomic sequence from patient E58-13 containing the insertion under study was amplified using KAPA HiFi HotStart PCR Kit (Kapa Biosystems), the specific patient genomic DNA and primers K1195 and K1196, and cloned into pBlueScript II KS+ vector between the *Bam*HI and *Hind*III restriction sites. Plasmid DNA was sequenced using K1207 to confirm the presence of the insertion. A single mutation in the PAM sequence was made to prevent cutting of this donor template plasmid using Quikchange mutagenesis kit (Agilent Technologies) and primers K1215 and K1216, and the change confirmed by sequencing. Next, this plasmid was amplified in linear fashion using primers K1219 and K1220, and the PCR product digested with *Dpn*I to remove any residual circular template plasmid. The antibiotic selection cassette (Puromycin resistance and truncated thymidine kinase) flanked by ITR sequences was amplified by PCR from PB-MV1 Puro-TK plasmid (Transposagen, Lexington KY) using primers K1217 and K1218, and ligated with the linearized plasmid (see above) at a TTAA region 78 bp upstream of the insertion using Gibson assembly Master Mix (New England Biolabs). NEB® 5-alpha Competent *E. coli* cells were transformed with 2 μl of the assembly reaction product. Plasmid DNA from 10 colonies were isolated and sequenced using primer K1217 to confirm correct assembly.

Genome editing using sgRNA and donor plasmids: For in vitro replication of InDels found in kidney disease patients, cultured human podocytes derived from a single cell were transfected by electroporation (Biorad laboratories, Hercules CA; Gene Pulser Xcell™ Electroporation System, 0.2 cm cuvette, square wave mode, 150 V and 10 millisecond pulse) with the CRISPR/Cas9 vector containing the specific sgRNA, and a donor plasmid containing the donor sequence and the antibiotic selection cassette. Following removal of non-transfected cells by incubation with 1 µg/ml Puromycin Dihydrochloride (Gibco) for 15 days, 10 µg of Excision-only piggyBac transposase expression vector (Transposagen) was transfected for scarless removal of the antibiotic selection cassette. Four days after transfection, cells were incubated with 2.5 µM ganciclovir (Sigma-Aldrich) to remove cells with residual truncated thymidine kinase activity. Single cells were picked, clones established, genomic DNA extracted using QIAamp DNA Mini Kit (QIAgen) and the target region PCR amplified using Platinum HiFi DNA polymerase (Invitrogen) and primers K1189 and K1188. PCR products were gel purified using QIAquick Gel Extraction Kit (QIAgen), cloned into pCR2.1 vector using TA cloning™ kit (Invitrogen) and the insert sequenced using the M13 Forward sequencing primer. Sequences were aligned with native podocyte genomic sequence and the donor template sequence by BLAST.

CRISPR A

Overall methods were identical to those for CRISPR B, with the exception of primers and oligonucleotides used, and the following site specific details: An 8 bp insertion (TGGATGGA) was introduced at Chr 8-122,304,094 - 122,304,095), and the sgRNA designed to recognize a specific site 73 bp upstream of the insertion site. While generating the donor plasmid, the patient specific genomic DNA (patient SF3) was cloned into the pBlueScript II KS+ vector between the *Spe*I and *Bam*HI sites. During Gibson assembly, the antibiotic resistance cassette was ligated with the linearized plasmid at a TTAA region 51 bp upstream of the insertion.

Agilent Custom capture and high throughput Illumina sequencing

A custom capture sequencing panel was created to isolate the genomic interval between *HAS2* and *ZHX2* on Chromosome 8. The target interval was uploaded to the SureDesign website for Agilent SureSelect capture probe design and synthesis (Agilent Technologies, Santa Clara CA). Genomic DNA library preparation and interval capture was done using the QXT SureSelect kit as per the manufacturer's instructions (Agilent Technologies). The resulting DNA libraries were quantitated by QPCR (Kapa Biosystems, Wilmington MA) and sequenced on the Illumina HiSeq 2500 or NextSeq 500 with paired end 100bp sequencing following standard protocols. Approximately 15 million sequences were obtained per reaction. FASTQ file generation was done using bcl2fastq converter from Illumina (Illumina, Inc., San Diego CA). Paired Illumina sequences compared with hg38 database (GRCh38.p13 Primary Assembly) using CLC Genomics software (Version 12, Qiagen, Venlo, the Netherlands). Insertion and deletions of 3 bp size or larger and a minimum of 20 sequence reads were selected for analysis. Fisher test comparison of insertions and deletions in study and control subjects was exported in Excel format, followed by software assisted and manual exclusion of all insertions and deletions present in controls. Next, FASTQ sequences were input into BWA (https://bio-bwa.sourceforge.net/) and mapped to hg38 to generate BAM files. The BAM files were input into PICARD (https://broadinstitute.github.io/picard/), and "build BAM index" tool used to generate the BAI file. Only insertions and deletions that were subsequently confirmed using BAM/BAI files on the IGV browser software (Broad Institute, Boston MA) were included. Establishment of homozygosity required presence of the InDel in over 85% of sequences, and subsequent confirmation by IGV. Minor discrepancies (1-2 base pair position differences) in the site of the insertion or deletion were occasionally noted between the two software and were resolved by Sanger sequencing while designing CRISPR Cas9 studies. All genomic numbering is based on hg38 and CLC Genomics software.

Hypothetical projection of Slc22a22 location in the human genome

BLAST based margins used the two peripheral parts of the mouse gene that matched with the human genome. BLAST and size based projections extended the BLAST based margins to the size of the mouse gene at either end.

STAT5, STAT6 and NFκB pathway studies in animal models

BALB/c and *BALB/cJ* mice (n = 3 mice per group) were injected with normal saline or Cocktail D dose 3X and euthanized at 15, 30 and 60 minute time points. Mice were perfused with protease inhibitors (Thermo Fisher Scientific, catalog number: A32953) and phosphatase inhibitors (Thermo Fisher Scientific, catalog number: A32957) via the left ventricle injection prior to euthanasia. For qualitative studies, total protein was extracted with RIPA buffer (Thermo Fisher Scientific, catalog number: 89900) from random sections of liver, heart and kidney in the presence of protease and phosphatase inhibitors to confirm activation of STAT5 (STAT5 and pSTAT5), STAT6 (STAT6 and pSTAT6) and NFκB (p65 and phospho-p65) pathways. For quantitative studies, nuclear and cytosolic fractions were separately extracted from correlated sections from these organs for each mouse separately using the Nuclear Extraction Kit (Novus Biological, Centennial CO, USA, cat # NBP-2-29447). Western blots for nuclear expressed protein Lamin B1 were conducted on both fractions to confirm predominant expression in the nuclear fraction. Western blot for GAPDH was conducted to confirm presence in both fractions. For relative quantitation by Western blot, pSTAT proteins were expressed as a ratio with Lamin B1 in nuclear fractions and the corresponding STAT protein in cytosolic fractions. Both ratio components were always scanned from the same non-saturated film image, and densitometry conducted using Bio-rad Image Lab 6.1 software with manual detection of close cropped bands, background subtraction, band identification, and adjusted total lane volume calculation. Antibodies against the following proteins were purchased: STAT5 (D2O69, 1:500), p-STAT5 (D47E7, 1:1000), STAT6 (D3H4, 1:500), P-STAT6 (D8S9Y, 1:1000), NF-κB p-65 (D14E12,

1:1000), P-NF-κB P-p-65 (S536, 1:1000), GAPDH 14C10, 1:20,000), all from Cell Signaling Technology, Inc. Danvers MA, USA; Lamin B1 (ab16048, 1:5,000, Abcam); Donkey anti Rabbit IgG HRP (1:20,000, Jackson Laboratories)

In vitro STAT6 signaling studies

Wild-type (precursor of CRISPR modified podocytes) and CRISPR-B podocytes were grown in RPMI 1640 media (Life Technologies catalog number 11875-085) containing heat-inactivated 10% fetal bovine serum, 1% Insulin-Transferrin-Selenium (ITS-G, Thermo Fisher Scientific - catalog number 41400045) and 1% Penicillin-Steptomycin (Thermo Fisher Scientific, catalog number 15140122) at 33^0C. Cells were sub-cultured and 50,000 cells/dish were seeded on 10cm culture dishes at 37^0C for 3 days. Next, culture media were exchanged with RPMI 1640 containing heat-inactivated 0.2% FBS and 1% Penicillin-Steptomycin. After 24hr, cells were treated with Cocktail C or Common Cold Cocktail (X/100,000) for 10, 20 and 30min. Proteins were isolated with RIPA buffer containing protease and phosphatase inhibitors (10ml of RIPA buffer contained 1 tablet each of protease and phosphatase inhibitor). Protein concentration was assessed using the Bradford protein assay. The following antibodies were used for Western blot: anti-pSTAT6 (1:500); anti-STAT6 (1:500).

Human plasma from COVID-19 and control patients for IL-4Rα assay

Human plasma 100 µL aliquots were obtained from the following sources (a) De-identified hospitalized COVID-19 patient samples from the Rush University COVID-19 Registry and Biorepository. (b) De-identified hospitalized COVID-19 patient samples from the Rush University COVID-19 Registry and Biorepository, selected for presence of proteinuria. (c) De-identified plasma samples that were age, sex and race matched to group a, purchased from Zenbio (Durham NC, USA)
